# Molecular Complexity Constrained Early Amino Acid Recruitment into the Genetic Code

**DOI:** 10.1093/gbe/evag012

**Published:** 2026-01-20

**Authors:** Syeda Ameena Hashmi, Hamed Chok, Ricardo Cabrera, Celia Blanco

**Affiliations:** Blue Marble Space Institute of Science, Seattle, WA 98104, USA; Blue Marble Space Institute of Science, Seattle, WA 98104, USA; Laboratorio de Bioquímica y Biología Molecular, Departamento de Biología, Facultad de Ciencias, Universidad de Chile, Ñuñoa, Santiago 7800003, Chile; Blue Marble Space Institute of Science, Seattle, WA 98104, USA; Centro de Astrobiología (CAB), CSIC-INTA, Torrejón de Ardoz, Madrid 28850, Spain

**Keywords:** genetic code evolution, molecular complexity, graph theory applications, amino acid chronology, molecular evolution

## Abstract

Previously proposed chronologies of amino acid incorporation into the genetic code rely on consensus rankings derived from prebiotic synthesis experiments, biosynthetic pathways, or genomic trends. However, the role of intrinsic molecular properties in shaping amino acid recruitment remains largely underexplored. In this study, we reconstruct a complexity-based amino acid chronology by integrating 16 molecular complexity metrics from chemical graph and information theory. Unlike approaches influenced by environmental variability, detection biases, or the evolutionary constraints of genome-based chronologies, our method provides a perspective on amino acid incorporation independent of these factors. Instead of imposing a linear ranking, we derive a minimum spanning tree capturing complexity-based relationships between amino acids. The resulting hierarchy places structurally simple amino acids in basal positions, while biosynthetically complex residues appear later, aligning with existing prebiotic and genomic chronologies. Furthermore, amino acids positioned closer in the complexity space exhibit significantly greater mutational connectivity than expected by chance, suggesting that molecular complexity reflects underlying structural considerations that constrained the genetic code's evolutionary pathways. This supports the idea that the code evolved not only to maintain biochemical stability but also to facilitate complexity-preserving substitutions, ensuring smooth adaptive transitions while minimizing energetic cost differences. Additionally, molecular complexity significantly correlates with amino acid enrichment in LUCA's inferred proteome, reinforcing its role as a fundamental constraint on early protein evolution. Our approach, rooted in intrinsic molecular properties rather than external contingencies, offers new insights into the constraints shaping the genetic code and expands the scope for identifying universal principles of biochemical evolution.

Significance The evolutionary order of amino acid incorporation into the genetic code has traditionally been inferred from criteria tied to structure-function relationships, evolutionary history, or biochemical pathways. Here, we introduce a novel framework based on intrinsic molecular complexity, independent of the chemical environment context. Amino acids closer in complexity are more frequently connected by single-point mutations, suggesting that molecular complexity constraints influenced substitution patterns during genetic code evolution. Furthermore, our minimum spanning tree reconstruction reveals that amino acid incorporation followed a complexity-constrained progression that maximized mutational accessibility. Notably, the complexity-derived chronology aligns closely with amino acid usage in LUCA and performs better than previously proposed chronologies. These findings provide a new dimension for understanding the genetic code's emergence and may inform the search for alternative biochemistries beyond Earth.

## Introduction

The emergence of life involved successive transitions toward greater molecular and organizational complexity, from simple inorganic chemistry to fully living systems. Molecular complexity, in particular, has long been a topic of interest for understanding the origin and evolution of life, yet the challenge lies in defining and quantifying it in ways that are both biologically meaningful and applicable across evolutionary timescales (Spitzer et al. 2015; Böttcher 2018; Kalambokidis and Travisano 2024).

In the last half-century, numerous efforts have been made to quantify molecular complexity by introducing new frameworks and metrics; however, defining the concept remains challenging. Although human perception often dictates which molecules appear “more complex”, this impression does not translate into a single, universal metric of molecular complexity (Oprea and Bologa 2023). As Steven Bertz noted in 1980: “*Synthetic chemists have been defining a ‘complex molecule’ in the way that many people define art: they know it when they see it*” (Bertz 1981). Early work in the field laid the foundation by modeling molecular structures as graphs, measuring connectivity, branching, and symmetry, while more recent approaches have expanded to include chemical information related to the atomic environments, like hybridization, chirality or bond types (Wright and Sarpong 2024). Alternative approaches such as collective intelligence (Li and Eastgate 2015), crowdsourcing (Sheridan et al. 2014) or fractal dimension (von Korff and Sander 2019) have also been proposed. Similar frameworks have been used to analyze amino acids complexity in meteorites through physicochemical and electronic properties (Da Pieve 2019), while other studies have examined structural and informational aspects of complexity, demonstrating its significance as a biologically relevant measure across various molecular systems (Nören-Müller et al. 2006; Schuffenhauer et al. 2006). Over time, different approaches have also been developed to allow for direct comparisons across a spectrum of biomolecules and even life-like systems (Böttcher 2016, 2018; Sharma et al. 2023).

In the context of life's emergence, molecular complexity can be thought of as the interplay of structural and informational richness in a molecule; an interplay that likely influenced which molecules took on the earliest roles in biological systems. Recent theoretical perspectives propose that such complexity not only reflects a molecule's architecture but may also encode the steps required to construct it, linking structural richness to both functional potential and historical constraints (Sharma et al. 2023). This broader view supports the idea that molecular complexity could have played a foundational role in the emergence of coding and replication (Dufton 1997; Mayer 2020; Kalambokidis and Travisano 2024), although it remains unclear how (or even if) it shaped the order in which amino acids were incorporated into the genetic code.

Attempts to establish a chronology or timeline for amino acid incorporation have traditionally relied on broad evolutionary and biochemical clues. For instance, pioneering studies like those of Trifonov built amino acid chronologies based on a variety of factors such as amino acid biosynthesis pathways, thermostability, and prebiotic abundance, along with a few criteria related to molecular complexity (Trifonov 2000, 2004). While informative, this approach has been debated, with critiques pointing to its reliance on diverse, sometimes qualitative factors that lack consistency across criteria (Fried et al. 2022; Zhao et al. 2022; Wehbi et al. 2024). However, a similar timeline can be inferred using only measurable amino acid concentrations in prebiotically plausible environments, following a more empirically grounded approach (Higgs and Pudritz 2009). Notably, that study found that the earliest amino acids tend to have the lowest free energies of formation, supporting the idea that the most thermodynamically accessible (and likely the simplest) entered the genetic code earlier.

Complementary to these chronology-based efforts are broader theories of genetic code evolution, such as environment-first models (Wong 1975) and coevolutionary frameworks centered on metabolic expansion (Wong 1975; Di Giulio 2008). Despite their conceptual differences, all these approaches converge on a shared qualitative observation: amino acids appear to have been incorporated into the genetic code in a general order of increasing structural complexity. This widely recognized yet unquantified pattern provides the conceptual motivation for the present study.

This raises a fundamental question: Could molecular complexity alone serve as a basis for establishing an amino acid chronology? If a complexity-based order aligns with existing amino acid chronologies, it would suggest that gradual increases in molecular complexity constrained the space of possibilities during the evolution of the genetic code. Here, we investigate how genetically coded amino acids relate to one another in terms of molecular complexity by integrating different metrics from graph theory and information theory frameworks. Previous chronologies typically impose a linear ranking (total order) of amino acid addition, sometimes grouping residues into broad batches (Trifonov 2000, 2004; Higgs and Pudritz 2009; Liu et al. 2010; Zhao et al. 2022; Wehbi et al. 2024), we construct a “complexity tree’ to model the potential chronological order of amino acid incorporation. Our approach preserves the underlying relationships between amino acids (e.g. branches of different depths), revealing patterns that a simple averaged rank succession would obscure. Strikingly, our complexity-derived chronology aligns closely with previously proposed amino acid chronologies (Trifonov 2000, 2004; Higgs and Pudritz 2009) and with LUCA's inferred usage (Wehbi et al. 2024). This alignment suggests that molecular complexity may not only describe the structural and functional aspects of amino acids but might have also imposed biochemical constraints during the early evolution of the genetic code. Besides supporting existing theories, our approach also offers a framework that positions molecular complexity as a fundamental organizing factor in the evolutionary history of biologically encoded information.

## Results

**Fig. 1.**
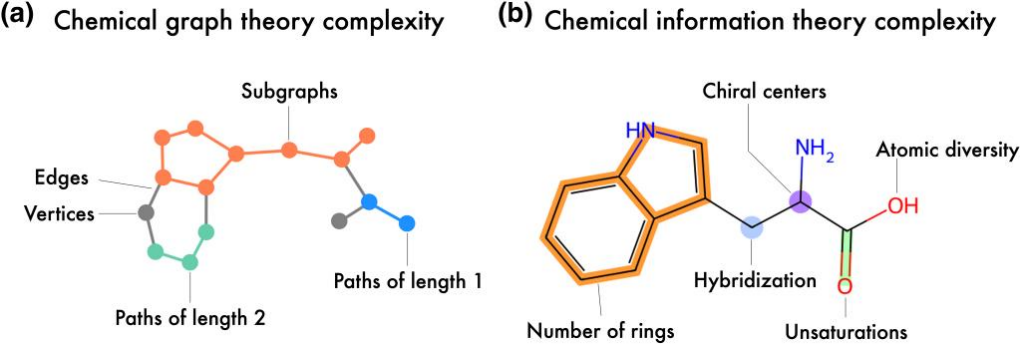
Representation of the two main frameworks used to quantify molecular complexity: (a) graph-theoretic and b) information-theoretic approaches. Tryptophan is depicted as both a graph representation and a molecular structure, illustrating some of the key features captured by each framework.

Over the past decades, two primary frameworks have emerged for quantifying molecular complexity: chemical graph theory and chemical information theory. Chemical graph theory represents molecular structures as abstract graphs, where atoms correspond to vertices and bonds to edges. This framework focuses on connectivity, topology, and structural organization, capturing molecular features such as branching patterns, ring systems, and paths of varying lengths. Through this lens, complexity emerges as a property defined by the spatial arrangement of atoms and the distribution of bonds. In contrast, chemical information theory focuses on the informational content encoded in molecular structures. This approach analyses the diversity and distribution of molecular features, considering factors like atomic environments, bonding classes, stereoisomerism, or symmetry. This framework treats molecules as carriers of information and focuses on how structural features contribute to their unique identity and functional diversity (Fig. 1). For a historical overview, see Ref. (Oprea and Bologa 2023); for a detailed review of metric types, see Ref. (Wright and Sarpong 2024). Together, these frameworks offer complementary perspectives: graph theory provides a geometric and topological foundation for understanding molecular structure, while information theory accounts for the diversity, redundancy, and asymmetry of structural components. Because both approaches derive directly from molecular structure rather than from empirical physicochemical properties, they provide a higher-order description that integrates multiple structural aspects within a single analytical framework. This makes them complementary—not redundant—to classical descriptors such as polarity or hydrophobicity, which capture only individual chemical attributes.

We selected 16 metrics to characterize the molecular complexity of amino acids: seven graph-theory-based (Appendix, Table S1) and nine information-based (Appendix, Table S2). The graph theory metrics include the Bertz/Hendrickson/Ihlenfeldt index (BHI) (Bertz 1981; Hendrickson et al. 1987), Balaban index (BAL) (Balaban 1982), log-transformed Walk Complexity (logWCX) (Ruecker and Ruecker 1993), Proudfoot index (PF) (Proudfoot 2017), Euclidean norm of the two symmetry-modified Zagreb indices (NSMM) (Gutman et al. 1975), Euclidean norm of the three Kappa indices (NK) (Hall and Kier 2007), and the Molecular Index (MI) (Jirasek et al. 2024). The information-based metrics include Whitlock's index (WH) (Whitlock 1998), Barone index (BAR) (Barone and Chanon 2001), Synthetic and Molecular Complexity Metric (SMCM) (Allu and Oprea 2005), normalized Bottcher Score (NBS) (Böttcher 2016), Fraction of Chiral Centers (FCC) (Lovering et al. 2009), Fraction of sp³-hybridized Carbons (FSP³) (Clemons et al. 2010), normalized Spacial-Score (nSPS) (Krzyzanowski et al. 2023), Minimal Graph Complexity (MGC) (Papentin 1982), and the Size/Complexity Score (SCS) (Dufton 1997). We used symmetry-modified versions of each metric where applicable (Walk Complexity WCX instead of Total Walk Count [Ruecker and Ruecker 1993] and symmetry-modified Zagreb indices) and normalized additive metrics to account for size effects (NBS and nSPS). Multi-component metrics were combined using the Euclidean norm (SMM1 and SMM2 combined into NSMM and K1, K2 and K3 combined into NK). The Walk Complexity was log-transformed (logWCX) to account for its exponential scaling with molecular size (e.g. for n-alkanes with *n* = 1 to 9, TWC grows from 2 to 6500) (Appendix, Fig. S1). Several other classical complexity metrics were not included, as they either represent outdated or less generalizable variants of those selected, or display undesirable scaling behaviors with structural features such as branching or cyclicity.

### Complexity as a Multidimensional Chemical Descriptor

While some metrics may partially reflect molecular weight, it is important to note that weight and complexity are fundamentally distinct properties, even if they are sometimes loosely conflated. While molecular weight is just the aggregate mass of a molecule, molecular complexity reflects the arrangement and organization of atoms in space. For example, pentane and glycine have nearly identical molecular weights (∼72 Da), yet only glycine contains the atomic configuration required for peptide formation and biological activity.

We computed molecular complexity values for the twenty proteinogenic amino acids using the set of 16 metrics encompassing both graph theory and information-theory approaches (Appendix, Table S3). We then compared the normalized values of all 16 metrics to the molecular weight of each amino acid to assess the relationship between molecular weight and complexity in the dataset. For all 320 datapoints (20 amino acids × 16 metrics), the correlation was weak (*R*^2^ = 0.18), indicating that molecular weight accounts for only a small fraction of the variation in structural complexity, and reinforcing the value of complexity as an independent and chemically meaningful descriptor (Appendix, Fig. S2).

A similar distinction applies to classical physicochemical properties such as hydropathy, polarity, or volume, which describe single molecular attributes that can increase or decrease independently depending on evolutionary pressures or environmental context. Physicochemical descriptors capture how molecules behave or interact in specific environments, whereas molecular complexity quantifies their intrinsic structural organization. The two are therefore related but not interchangeable: one is functional, the other structural. Individual physicochemical properties can fluctuate without implying directional progression, since evolution could favor amino acids with higher or lower polarity or volume depending on context. In contrast, structural complexity accumulates through the elaboration of molecular architecture and provides a coherent quantitative axis along which molecular diversification can be ordered.

This cumulative character makes complexity both quantitatively and qualitatively different from individual properties; it provides a natural expectation of incremental change, consistent with the progressive structural diversification observed in biochemical evolution. Although definitions vary, they are formalized, reproducible, and empirically grounded—no less objective than composite physicochemical descriptors such as hydrophobicity, whose numerical scales depend on convention yet capture a real and measurable property.

### Pairwise Comparative Analysis of Frameworks

**Fig. 2.**
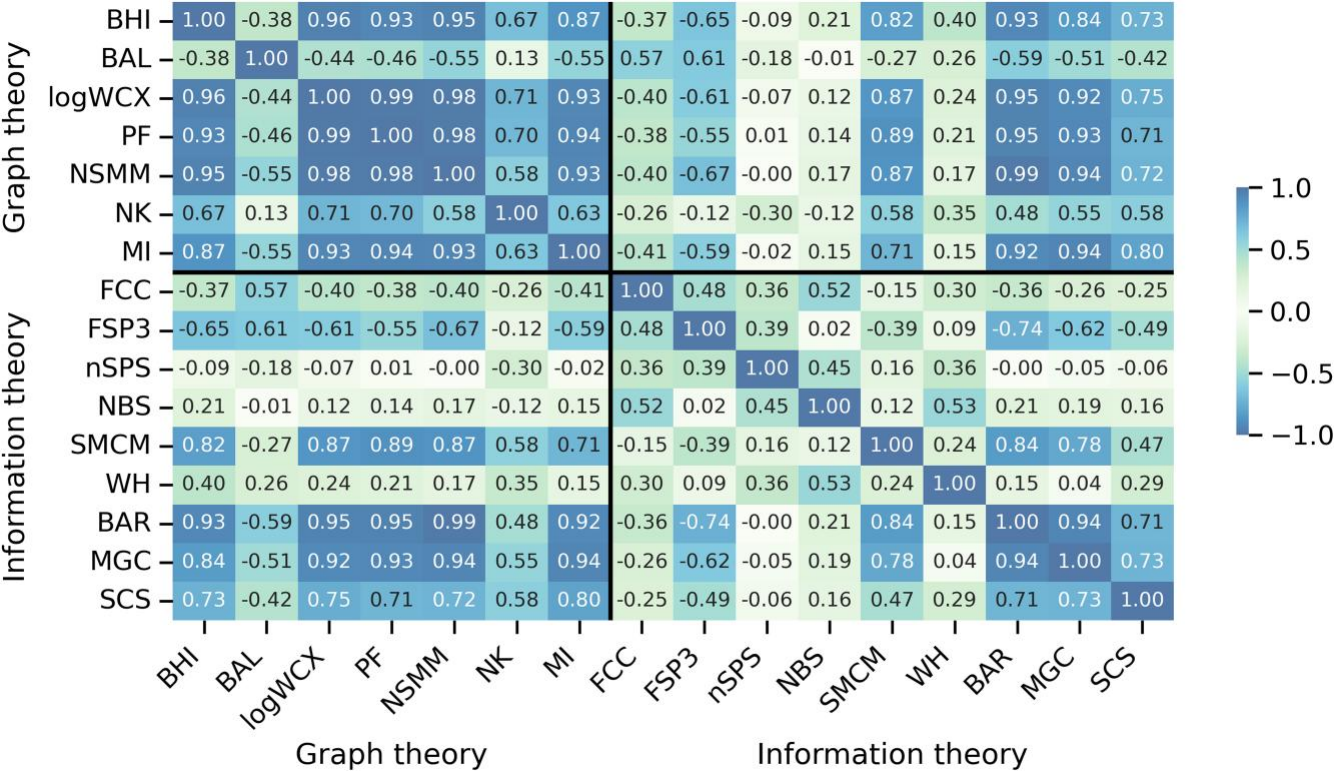
Symmetric matrix showing Pearson correlations among the 16 molecular complexity metrics included in the final analysis. These include scale-adjusted metrics (logWCX, nSPS and NBS), and composite metrics computed as Euclidean norms (NSMM from SMM1 and SMM2; NK from K1–K3). Black, thick lines represent the division between graph theory-based metrics and information theory-based metrics.

We performed pairwise Pearson correlation analysis across all 16 metrics to better understand how they relate to one another (Fig. 2 and Appendix, Fig. S3). As expected, graph-theory-based metrics exhibit strong positive correlations, indicating that they capture similar aspects of molecular connectivity and size. In contrast, information-theory-based metrics, which emphasize localized atomic environments and patterns, show weaker or even negative correlations with both graph-theoretical metrics and among themselves. This divergence suggests that graph theory metrics provide a more unified view of molecular complexity, while information-theory metrics capture orthogonal and fine-grained structural features. It is worth noting that this pattern may be specific to the canonical amino acids and may not generalize across other molecular classes. The lack of full agreement between and within frameworks reinforces the idea that no single metric fully defines molecular complexity, motivating the use of an integrated multimetric approach for a more comprehensive characterization. Each metric captures a different aspect of molecular architecture, and it is their integration that enables a multidimensional comparison of amino acid complexity.

### Mutation Accessibility Reflects Proximity in the Complexity Space

If molecular complexity influenced –or at least paralleled– the structure of the genetic code, then amino acids with similar complexity profiles may have been more likely to substitute for one another via single-nucleotide mutations. Each amino acid, *a_i_*, is represented as a vector in a multidimensional metric space, where molecular complexity is encoded as a set of real-valued, numerical features derived from the 16 selected metrics. In this metricized vector space, the amino acids form a point cloud—a collection of discrete positions whose pairwise distances, *d_ij_*, reflect the similarities and differences between amino acids *a_i_* and *a_j_*. We computed these distances using the Euclidean norm in the original 16-dimensional space, preserving the full resolution of the complexity profiles. The resulting distance matrix was then used to evaluate whether amino acid pairs connected by single-nucleotide mutations in codon space tend to lie closer together in complexity space.

**Fig. 3.**
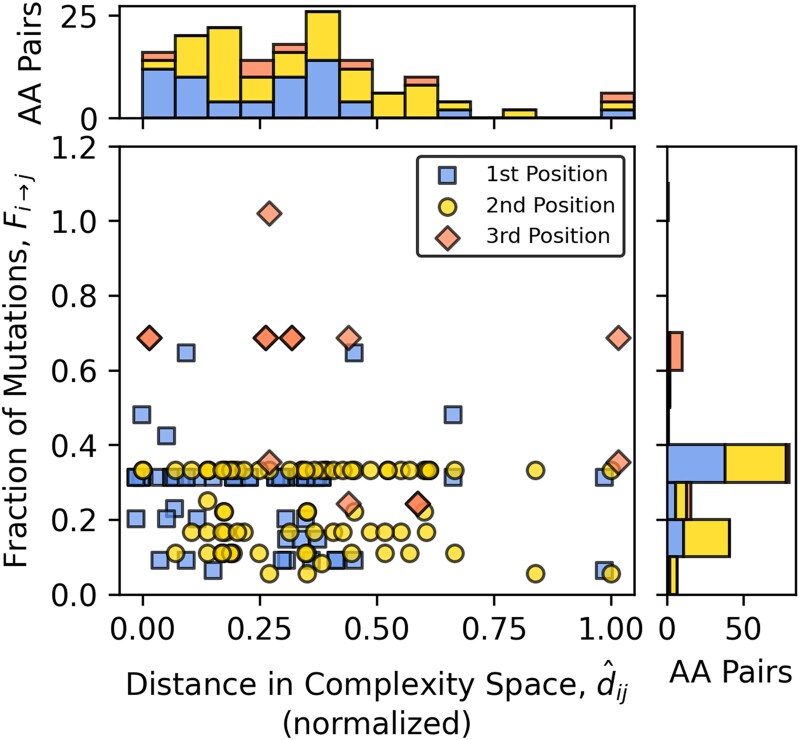
Relationship between normalized molecular complexity distance, $\hat{d_{ij}}$, and the fraction of possible single-point mutations (*f_i_*→*_j_*), for amino acids (*a_i_*, *a_j_*). The central scatterplot shows all amino acid pairs, with mutations separated by codon position: first (blue squares), second (yellow circles), and third (orange diamonds). Points are slightly offset (0.2 in x, 0.02 in y) to reduce overlap. Marginal histograms display the distributions of normalized distances (top) and mutation fractions (right) for each codon position, using the same color scheme as the scatter.

For each amino acid pair, *a_i_* and *a_j_*, we calculated the fraction of all possible single-nucleotide substitutions in the codon space that convert a codon for *a_i_* into a codon for *a_j_*, denoted *F_i_*→*_j_*. We then compared these mutation fractions to the corresponding normalized pairwise distances in the molecular complexity space, $\hat{d_{ij}}$ (the raw distances $d_{ij}$ were min–max normalized to the [0,1] range so that pairs with $\hat{d_{ij}}\approx 0$ are the closest and those with $\hat{d_{ij}}\approx 1$ are the furthest in complexity). Notably, amino acid pairs with the highest mutation fractions tend to be closer in complexity space (Appendix Fig. S4). We computed *F_i_*→*_j_* separately for substitutions occurring at the first, second, and third codon positions to explore position-specific effects. The third position shows consistently higher mutation fractions and the strongest inverse correlation with molecular complexity distance (Fig. 3 and Appendix, Fig. S5). The first position follows a similar but weaker pattern, while the second position exhibits lower mutation fractions with no clear trend across the complexity distance range. These results indicate that the genetic code's structure aligns mutational accessibility with similarity in molecular complexity, a pattern consistent with error-minimization principles (Haig and Hurst 1999; Higgs 2009) but derived here from intrinsic structure rather than physicochemical properties. This alignment should not be interpreted as causal, but rather as evidence that amino acids accessible through single mutations tend to be similar in overall structural complexity.

To test whether graph- and information-theory frameworks yield comparable higher-order representations, we compared their pairwise distance matrices and found significant divergence between them (Appendix, Fig. S6).

### Complexity Progression From Minimum Spanning Tree Analysis

**Fig. 4.**
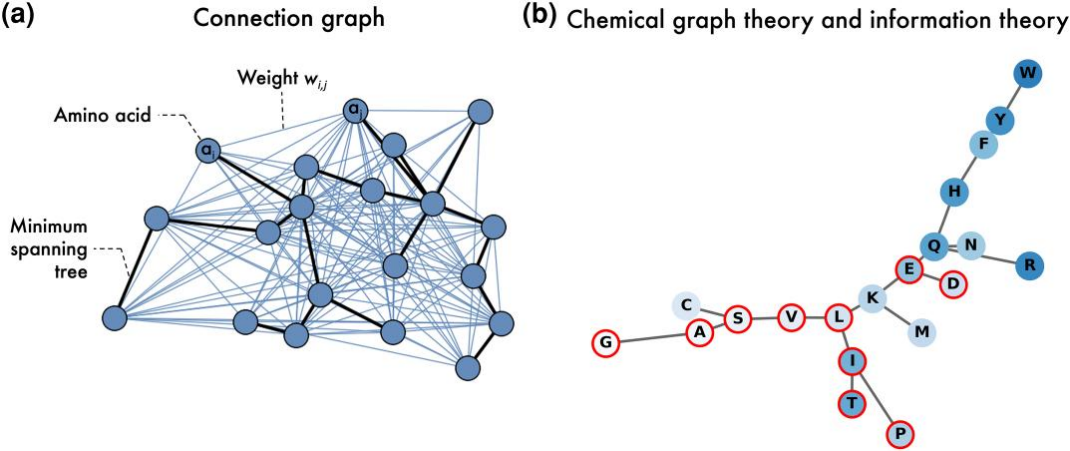
a) Connection graph based on simplicial complex representation. Nodes represent amino acids (*a_i_*, *a_j_*) and edge weights (*w_i,j_*) correspond to distances (*d_i,j_*), in the metricized vector space. Black edges illustrate the MST, corresponding to the most parsimonious (i.e. shortest total distance (also minimum total weight)) connectivity among all amino acids. b) MST using the full set of 16 complexity metrics embedded over the lower-dimensional space. Amino acids are color-coded according to Trifonov's proposed chronology (Trifonov 2004), with lighter colors indicating “early’ amino acids and darker colors representing “later’ ones (Longo and Blaber 2012). Nodes with thick, red edges denote amino acids supported by prebiotic evidence from meteoritic analyses, simulated prebiotic chemistry, and hydrothermal vent experiments (Longo and Blaber 2012). Edges length is made proportional to weight in the tree connections (i.e. original distance); however, distances between nodes that are not directly connected do not necessarily reflect their original pairwise distances in the metric space.

We analyzed the topological relationships among amino acids in complexity space to infer a possible chronology of their incorporation into the genetic code. To do this, we characterized the structure of the complexity point cloud using a combination of dimensionality reduction and topological and graph-based representations. The original 16-dimensional metric space was first embedded into a 7-dimensional Euclidean space using the multidimensional scaling (MDS) algorithm (Kruskal 1964), which preserved pairwise distances with a reconstruction error of less than 1.56% on average and below 5.95% at the 95th percentile (Appendix, Fig. S7). Because several of the complexity metrics are correlated (Fig. 2), this embedding captures their shared covariance structure, allowing the 16-dimensional space to be represented in seven dimensions with minimal information loss. This lower-dimensional embedding allowed for a more efficient data representation and subsequent computation while recovering the inherent data dimensionality. We then constructed a Vietoris-Rips (VR) simplicial complex (Vietoris 1927; Hausmann 1996) that was ultimately reduced to a point proximity graph structure, linking amino acids within a predefined distance threshold. The VR complex recovers the underlying data topology (or less formally, the neighborhood information). Such inferred topological information was precisely what was used to generate the aforementioned point-to-point connection graph (Fig. 4a). Finally, we applied a minimum spanning tree (MST) algorithm (Prim 1957) to obtain the most parsimonious set of edges connecting all 20 amino acids based on pairwise complexity distances. This does not imply that the genetic code evolved through a greedy optimization process. Rather, the MST provides a principled structure for inferring a potential order of amino acid incorporation under the assumption of incremental complexity. If the MST-based chronology produces biologically coherent outcomes, it supports the plausibility of this framework; if not, the assumption can be rejected, which would also be a meaningful outcome. As shown below, the resulting greedy linkage of amino acids (i.e. the MST) yields interpretations that align with current evolutionary consensus while providing new insights on the structural relationships among amino acids. We computed the MST using Prim's algorithm (Prim 1957), which yields a single, well-defined optimum tree because all pairwise distances in the graph are unique. This produced a non-directional tree that preserves structural relationships among amino acids without imposing a linear order (Fig. 4b).

The structure of the complexity-derived tree based on the full set of molecular complexity metrics reveals a clear pattern in how amino acids are arranged based on their physicochemical properties (Appendix, Fig. S8). Aliphatic and hydroxyl-containing amino acids tend to cluster toward one end of the tree, while acidic, amidic, basic, and aromatic amino acids are positioned further away. Considering Gly (G) as the simplest, the amino acids appearing at the lower end of the complexity spectrum include Ala (A), Ser (S), Cys (C), Val (V), Leu (L), Ile (I), Thr (T), and Pro (P), which are primarily small and aliphatic or hydroxylated (except for C). Acidic (Asp (D), Glu (E)), basic (Lys (K), Arg (R), His (H)), and amidic (Asn (N), Gln (Q)) amino acids occupy intermediate positions, as well as Met (M). The aromatic residues (Phe (F), Tyr (Y), and Trp (W)) are positioned toward the highest end.

Notably, except for some outliers (see Discussion Section), this distribution mirrors the widely proposed distinction between simpler amino acids that are thought to have been available in early biochemical systems and more complex amino acids that required advanced biosynthetic pathways (Trifonov 2004; Longo and Blaber 2012). This correspondence reinforces the long-recognized trajectory of increasing amino acid complexity inferred from both prebiotic chemistry and biosynthetic analyses (Wong 1975; Trifonov 2004; Di Giulio 2008; Higgs and Pudritz 2009), here recapitulated directly from structural data without invoking specific mechanistic assumptions. We also applied the full three-step pipeline (MDS, simplicial complex construction, and MST generation) to each framework independently and found that the resulting tree structures differ substantially, confirming that each framework captures distinct features (Appendix, Fig. S9).

### Complexity-derived Tree Exhibits Enriched Mutational Connectivity

A key feature of a meaningful amino acid chronology is not just its internal structure, but its ability to reflect biologically plausible mutational paths. If a complexity-based tree captures underlying evolutionary constraints, then its edges should align with accessible mutational transitions in the genetic code. We found that the complexity-derived tree exhibits a significantly higher number of single-point mutations connecting its edges than expected under random amino acid arrangements. To test whether this connectivity could arise by chance, we randomized the assignment of amino acids to the tree nodes (while preserving the tree's topology) and recalculated the number of connecting mutations across *N* = 10^5^ trials. Compared to these randomized trees, the complexity-derived tree consistently showed greater mutational connectivity, both in terms of the absolute number of mutations and the fraction of possible transitions used (Table 1). This enrichment does not imply that complexity determined mutational patterns, but rather that the observed arrangement aligns with the inherent structure of the genetic code.

**Table 1:** Mutational connectivity in complexity-derived versus randomized minimum spanning trees

	No. of mutations		Fraction of mutations	
	Complexity-derived MST	95% C.I. for Randomized MSTs (*N* = 105)	Complexity-derived MST	95% C.I. for Randomized MSTs (*N* = 105)
First position	20	8.290 ± 0.029	0.104	0.051
Second position	14	8.800 ± 0.025	0.073	0.054
Third position	8	2.510 ± 0.018	0.042	0.015
Total	42	19.600 ± 0.038	0.073	0.040

Number and fraction of single-point mutations in the complexity-derived minimum spanning tree (MST) and randomized MSTs (*T* = 10^5^). The observed values exceed the 95% confidence intervals (C.I.) of the randomized trees, proving a structured amino acid progression that maximizes mutational accessibility. For fractional values, 95% C.I.s were <0.001 and therefore are not displayed.

This result is consistent with expectations based on random amino acid pairings. In a randomly ordered path graph (i.e. a linear tree with no branches), the expected number of single-point mutations connecting adjacent amino acids is 8.32, 8.59, and 2.89 for the first, second, and third codon positions, respectively (see Methods Section). The corresponding observed values in the complexity-derived tree (20, 14, and 8) substantially exceed these expectations, reinforcing the idea that the arrangement of amino acids in the tree coincides with mutational connectivity beyond what would be expected by chance.

This finding is further supported by an independent comparison using a codon substitution matrix that incorporates transition/transversion biases and position-specific mutation rates (Ali and Borah 2021). The average distance between adjacent amino acids in our tree (5.93) is notably lower than the overall average calculated across all amino acid pairs (7.76) (Appendix, Fig. S10), providing additional evidence that the tree structure preserves biologically plausible mutational proximity.

### Low-complexity Amino Acids are Enriched in LUCA's Proteome

**Fig. 5.**
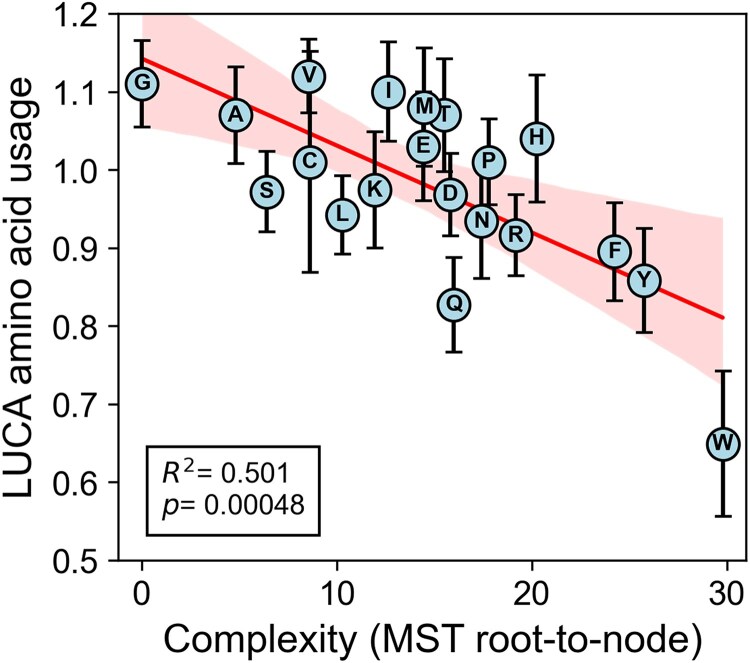
Correlation between molecular complexity, defined as root-to-node (Appendix, Table S4) and LUCA amino acid usage. Error bars represent 95% confidence intervals derived from reported standard errors (Wehbi et al. 2024). The red line shows linear regression fit, with shaded area indicating the 95% confidence interval of the fit.

Existing amino acids chronologies have been inferred using ranking-based methods, where amino acids are ordered by specific criteria and their ranks are averaged (Trifonov 2000, 2004; Higgs and Pudritz 2009; Liu et al. 2010; Zhao et al. 2022; Wehbi et al. 2024). While useful for broad comparisons, such methods do not capture the relative distances between amino acids in the metric space. Instead of averaging the absolute ranking derived from the raw data, we computed the cumulative distance from the root (considered as the less complex amino acid, Gly) to each other amino acid in the complexity-derived tree, preserving structural relationships. LUCA's amino acid usage is treated here as an empirical reference for comparison, not as evidence for the environmental origin of the genetic code. Amino acids with lower molecular complexity, as measured by root-to-node distance in the MST (Appendix, Table S4), exhibit significantly higher enrichment in LUCA's inferred usage (Wehbi et al. 2024) (*R*^2^ = 0.5013, *P* = 0.0005) (Fig. 5). This correlation is much higher than those between LUCA's usage and Trifonov's consensus chronologies (*R*^2^ = 0.2786, *P* = 0.0167 for Trifonov's 2000 order (Trifonov 2000), and R^2^ = 0.3000, *P* = 0.0124 for Trifonov's 2004 order (Trifonov 2004)) (Appendix, Fig. S11). We focused our analysis on LUCA-level amino acid usage as this stage most directly reflects the canonical set of proteinogenic amino acids integrated into genetic coding. Pre-LUCA usage, while informative about early metabolic expansion, may reflect broader chemical availability and is less constrained by coding structure. The analysis shows a weaker correlation between complexity and pre-LUCA usage (*R*^2^ = 0.331), consistent with this interpretation.

To compare to existing chronologies using an equivalent method, we computed an overall order using a rank-based approach (Appendix, Tables S5 and S6). Notably, LUCA's usage predictions are considerably weaker using the average complexity rank (R^2^ = 0.29344, *P* = 0.01363) (Appendix, Fig. S12a and b). The same is observed for Uniprot composition (root-to-node: R^2^ = 0.40718, *P* = 0.00247; average rank: R^2^ = 0.29907, *P* = 0.01259) (Appendix, Fig. S12c and d). The lower correlation between complexity and Uniprot usage (UniProt Consortium 2025), compared to LUCA, likely reflects the cumulative effects of billions of years of evolutionary change, including selective pressures, functional specialization, and genomic drift, which obscure early constraints.

## Discussion

The idea that small, incremental increases in molecular complexity influenced the evolutionary order of amino acid incorporation into the genetic code is not new (Dufton 1997), although it has remained relatively underexplored. The high codon degeneracy and widespread use of simpler amino acids in proteins suggest that the code evolved to favor structurally less complex residues, likely to enhance biosynthetic efficiency and reduce the risk of disruptive mutations (Dufton 1997). This general pattern is consistent with both environment-first models, which propose that simple amino acids were initially supplied by prebiotic synthesis (Wong 1975; Higgs and Pudritz 2009), and with frameworks that attribute amino acid incorporation to stepwise metabolic innovation (Di Giulio 2008). Although the number of biosynthetic steps offers a biologically grounded measure of metabolic elaboration in contemporary metabolic routes, it does not necessarily capture intrinsic molecular complexity, since pathways can follow non-minimal or branching routes shaped by historical contingencies. Our framework complements these views by quantifying molecular elaboration directly from structure, independent of metabolic or environmental assumptions, and by showing that the same progressive trend is captured when analyzed purely through molecular architecture.

Building on this conceptual foundation, we introduce a quantitative framework that links molecular complexity to early amino acid selection. Our analysis reveals that graph theory and information-theory frameworks capture complementary aspects of amino acid complexity. While raw metrics and distance matrices show strong internal consistency within each framework, they diverge significantly from each other, and their respective tree topologies reflect these differences. This lack of convergence highlights the absence of a universal definition of molecular complexity and motivates our integrated approach, combining both frameworks to obtain a more complete and chemically grounded representation.

We find that the genetic code tends to preserve molecular complexity across single-nucleotide substitutions, especially at the first and third codon positions. This pattern aligns with structural studies of ribosome-tRNA interactions, which show strict base pairing at the second codon site but relaxed specificity at the wobble position, supporting Crick's wobble hypothesis and the idea that the third position may have been less essential during early code evolution (Crick 1966; Jukes 1973; Hayes 1998; Ogle et al. 2003, 2001; Ogle and Ramakrishnan 2005). Because translation errors are more frequent than DNA mutations and disproportionately affect the first and third codon positions (Novozhilov et al. 2007), early selection pressures may have favored codon arrangements that preserve molecular complexity across substitutions. Indeed, the complexity-derived tree we construct is enriched for mutationally accessible transitions, as shown by two independent analyses: one using codon-level mutation counts across randomized trees and another based on a substitution matrix incorporating transition/transversion asymmetries and codon position biases. These findings suggest that the genetic code evolved not only under biosynthetic and functional constraints, but also to preserve complexity gradients and support mutational robustness. By minimizing disruptive substitutions and maintaining functional continuity, the code's structure likely facilitated smooth adaptive trajectories and reduced evolutionary bottlenecks, consistent with prior studies on genetic code robustness and neutral drift (Koonin and Novozhilov 2009; Tenaillon and Matic 2020; Rozhoňová et al. 2024).

If the genetic code preserves molecular complexity across substitutions, it is natural to ask whether molecular complexity also influenced the order in which amino acids were incorporated. The structure of the complexity-derived tree suggests that it did: simple, prebiotically abundant amino acids appear near the root, while biosynthetically complex residues are added later. This pattern is consistent with the significant enrichment of low-complexity amino acids in LUCA's proteome, supporting the role of molecular simplicity in early protein evolution. Early amino acids, such as Gly, Ala and Val, are chemical simple and stable, making them plausible candidates for primitive protein formation. Notably, the non-polar amino acids Leu, Ile and Val, exhibit low molecular complexity and were likely incorporated early into the genetic code, contributing to the formation of hydrophobic cores, a key feature of protein stability in aqueous environments and a precursor to functional tertiary structures (Longo and Blaber 2012). Hydroxyl residues Ser and Thr, also low in complexity, may have supported solubility and hydrogen bonding, and possibly stabilize helix–membrane interfaces, as suggested by the enrichment of small polar residues (Ala, Gly, Ser, Thr) at helix interfaces in membrane proteins (Eilers et al. 2002).

As complexity increases, acidic (Asp, Glu), basic (Arg, Lys, His), and amidic residues (Asn, Gln) introduced charge interactions and increased hydrogen-bonding capacity, supporting more elaborate folding and functional diversification (Jones et al. 2001; Treger and Westhof 2001; Kim et al. 2006; Blanco et al. 2018). These residues may have enabled folding under diverse environmental conditions: acidic residues supporting halophilic folding in high-salinity settings, and the broader group of charged and amidic residues contributing to mesophilic folding and structural stability under more moderate conditions (Longo et al. 2013). Their incorporation also likely reflects the emergence of more complex metabolic networks and enzymatic functions. Interestingly, however, our complexity-derived tree separates Lys and Arg, which often cluster together in analyses based on physicochemical properties such as charge or polarity. This distinction arises from their divergent molecular architectures: Lys is an aliphatic residue terminating in a primary amine, whereas Arg contains a branched guanidinium group with delocalized charge. A similar separation appears in the four-column theory for the origin of the genetic code (Higgs 2009), where Lys and Arg occupy different columns of the genetic code (3 and 4, respectively), and in Di Giulio's extension of the coevolution theory (Di Giulio 2008), where Arg requires more biosynthetic steps than Lys.

Aromatic amino acids (Phe, Tyr, Trp) appear last in the complexity tree, consistent with their high biosynthetic cost and specialized functions in catalysis, structural stabilization via π-stacking, and UV absorption (Bartlett et al. 2002; Biter et al. 2019; Kaiser et al. 2020). Their late incorporation likely reflects the emergence of metabolic networks capable of supporting their synthesis. Notably, the addition of aromatic and sulfur-containing residues such as Tyr, Trp, and Met may also have been driven by their antioxidant properties, which became increasingly important as rising atmospheric oxygen levels introduced oxidative stress (Bender et al. 2008; Berlett and Levine 2014; Schindeldecker and Moosmann 2015).

Our complexity-derived chronology outperforms Trifonov's consensus rankings in predicting LUCA usage (Wehbi et al. 2024). It also aligns well with multicriteria approaches that integrate prebiotic plausibility  (Trifonov 2004; Higgs and Pudritz 2009) (Table 2) and with theories of genetic code evolution grounded in prebiotic considerations (Higgs 2009; Higgs and Pudritz 2009). However, amino acids like Cys, Met, and Lys, exhibit low complexity but are rarely considered prebiotic. This discrepancy likely reflects limitations in early experimental frameworks rather than true absence. Sulfur-containing amino acids, such as Cys and Met, were excluded from early prebiotic experiments like Miller-Urey due to the absence of sulfur sources. However, later studies incorporating hydrogen sulfide (H_2_S) demonstrated Met synthesis (Van Trump and Miller 1972; Parker et al. 2011), and both amino acids have been detected in hydrothermal and UV-irradiation setups with H_2_S (Becker et al. 1974; Hennet et al. 1992), suggesting their prebiotic availability may have been underestimated. Lys presents a different challenge: it has not been detected in meteorites, potentially due to analytical biases such as degradation during derivatization, inefficient synthesis, or short half-life (Nuevo et al. 2008; Cleaves 2010; Raggi et al. 2016). Nonetheless, positively charged residues like Lys have been proposed as essential for nucleic acid binding in early peptides, implying functional necessity may have driven their early incorporation (Blanco et al. 2018); indeed, comparative genomic analyses show that proteins lacking basic amino acids are significantly shorter (34 to 111 residues), suggesting that their inclusion was key for structural complexity (McDonald and Storrie-Lombardi 2010). Recent phylogenetic reconstructions further indicate that Cys, Met, and Lys may have entered the code earlier than traditionally assumed, supporting the importance of sulfur metabolism and metal-catalyzed chemistry in early evolution (Wehbi et al. 2024).

**Table 2:** Comparison of amino acid chronologies from molecular complexity and previous studies

…	1	2	3	4	5	6	7	8	9	10	11	12	13	14	15	16	17	18	19	20
Molecular complexity (root-to-node; this study)	G	A	S	V	C	L	K	I	E	M	T	D	Q	N	P	R	H	F	Y	W
Multicriteria (Trifonov 2000)	G A	V D	P	S	E	L	T	R	N	K	Q	I	C	H	F	M			Y	W
Multicriteria (Trifonov 2004)	G	A	D	V	P	S	E	L	T	R	I	Q	N	H	K	C	F	Y	M	W
Meteorites, icy grains, atmospheric synthesis, hydrothermal synthesis, and other chemical syntheses (Higgs and Pudritz 2009)	G	A	D	E	V	S	I	L	P	T	K	F	R	H	N	Q	C	Y	M	W

Order of amino acid incorporation into the genetic code as inferred from our molecular complexity analysis (based on root-to-node distances in the minimum spanning tree), alongside other proposed chronologies.

Our approach also departs from traditional ranking-based methods (Trifonov 2000, 2004; Higgs and Pudritz 2009; Liu et al. 2010; Zhao et al. 2022; Wehbi et al. 2024), which assume a strictly linear progression of amino acid incorporation. These methods assign fixed ranks to each amino acid based on one or more criteria, disregarding the magnitude of differences between values. As a result, they may obscure meaningful structural or functional relationships and oversimplify the evolutionary process. In contrast, our framework treats molecular complexity as a continuous landscape and captures inter-amino acid relationships through a network-based representation rather than a rigid hierarchy. When applied to the same set of complexity metrics, this structural approach produces a substantially stronger correlation with LUCA's inferred amino acid usage than rank-based methods.

From an intuitive standpoint, in our view, there is no inherent reason to expect that abundance (whether prebiotic or in LUCA) should directly determine the order of amino acid incorporation. An amino acid may be environmentally abundant yet functionally redundant, or conversely, scarce but structurally indispensable. Similarly, usage frequencies in LUCA might reflect evolutionary outcomes rather than starting points. Although molecular weight has been shown to correlate with LUCA usage (Wehbi et al. 2024), it is worth reiterating that mass alone does not capture the architectural features that influence biochemical function or evolutionary accessibility. The same applies to thermodynamic arguments based on standard Gibbs free energies of formation (ΔG°) (Higgs 2009; Higgs and Pudritz 2009): although low-energy compounds are generally easier to synthesize, ΔG° values depend strongly on environmental parameters such as temperature, solvent composition, and redox state. If the genetic code evolved across different thermal or chemical regimes, as current evidence suggests, ΔG° would not represent a stable or universal criterion for ordering amino acids. Instead, we therefore propose a multidimensional, structure-aware view of molecular evolution. In that sense, a molecular complexity framework predicts that more complex amino acids will require biosynthetic or structural contexts that emerge later in evolution, making complexity a plausible constraint on the order of recruitment. Rather than a simple correlation, complexity reflects the increasing organizational capacity of evolving systems, an idea that resonates with recent theoretical proposals linking molecular structure to functional and historical constraints (Sharma et al. 2023).

The broader question of whether complexity tends to increase over evolutionary time has long been debated in biology, particularly in light of the interplay between contingency and necessity (McShea and Brandon 2010; Gould 2011; Moya 2015). While evolution proceeds through historically contingent events, there is also a passive expectation that complexity will accumulate over time, simply because it cannot fall below the minimum level where it started, a view famously described by Gould as a “left wall” of minimal complexity. For such a trend to be meaningfully assessed, however, one must first identify metrics that increase in tandem with evolutionary accessibility (Day 2012; Corominas-Murtra et al. 2018). Chemically grounded complexity measures that quantify intrinsic molecular architecture may offer a biologically meaningful way to evaluate how structural constraints influence evolutionary accessibility. If complexity constrains the transition from prebiotically available to biosynthetically produced building blocks during early evolution, then directional trends toward increasing complexity may emerge. This would not be the result of teleology, but rather a reflection of the expanding organizational potential of evolving systems.

While our framework offers new insight into early code evolution, several limitations and assumptions should be considered. First, the analysis is limited to the 20 proteinogenic amino acids, excluding non-biological alternatives that may have been relevant in prebiotic environments. Future studies incorporating non-canonical amino acids could provide further insights into the role of molecular complexity in shaping early biochemical evolution. Second, our approach assumes that the standard genetic code preserves meaningful information about early amino acid selection, though alternative coding schemes and lost evolutionary intermediates could have influenced early biochemical systems. It is also important to note that the complexity-derived MSTs do not necessarily reflect the precise temporal order of amino acid incorporation, as molecular complexity metrics do not account for all relevant pressures, such as folding dynamics or catalytic efficiency. Third, although molecular complexity is an intrinsic property of amino acids and independent of specific environmental contexts, its influence on genetic code evolution likely occurred in conjunction with other evolutionary and biochemical constraints. Future work integrating intrinsic complexity with environmental factors and intermediate coding stages may help clarify how these forces jointly shaped the genetic code. Finally, our approach primarily addresses the constraints shaping codon assignment during genetic code evolution, rather than the broader chemical transition from prebiotic to biotic systems. Although complexity likely influenced both, our MST reflects mutational and structural relationships within the coded set, not the chemical ease of synthesis per se.

Because molecular complexity arises from the intrinsic features of molecules and is not dependent on evolutionary context, it may offer a universal lens for understanding biochemical organization. If complexity influenced amino acid recruitment during the early evolution of life on Earth, similar constraints could apply to any chemically encoded system undergoing Darwinian evolution. This perspective could help identify generic patterns of molecular usage, offering a framework to interpret biosignatures and assess the plausibility of alternative biochemical systems.

## Materials and Methods

### Complexity Metrics Calculation

Each metric was computed using published definitions and implementations, with modifications as needed to match original formulations (Appendix, Tables S1 to S3). BHI was obtained from PubChem (Kim et al. 2023). BAL, K1, K2, K3, FCC, and Fsp3 were computed using RDKit in Python (Landrum et al. 2023). WCX was computed using a modified version of Molcomplex (Wright et al. 2024) (the code was modified to exclude the ½ factor, see Note (23) in ref. (Gutman et al. 2001), and to account for symmetry as per ref. (Nikolić et al. 2003)). PF was computed using AstraZeneca's complexity calculation tool (GitHub, n.d.). SMM1 and SMM2 were computed using a modified version of Mordred (Moriwaki et al. 2018) (the code was modified to account for symmetry, as per ref. (Nikolic et al. 2000)). MI was computed using AssemblyGo (Jirasek et al. 2024). nSPS was computed using the Spacial-Score (Krzyzanowski et al. 2023). NBS values were obtained from ref. (Böttcher 2016). SMCM values were computed using a modified version of Medchem (the code was modified to count only non-aromatic rings, as per ref. (Allu and Oprea 2005)). WS and BAR were computed using Medchem's original code. MGC values and SCS values were obtained from ref. (Papentin 1982) and ref. (Dufton 1997), respectively.

### Distance Matrix Computation

Pairwise distances between amino acids were computed in the full metric space to preserve the complete complexity profile of each residue. The input matrix $X\in R^{n\times p}$ contained autoscaled complexity values (mean-centered, unit variance) for n=20 amino acids across p molecular complexity metrics (16 for the full set, 7 for graph-based metrics, or 9 for information-based metrics). Distances were calculated using the Euclidean norm: $d_{ij}=\sqrt{\overset{p}{\underset{k=1}{\sum }}\;{\left(x_{ik}-x_{\;jk}\right)}^{2}}$, where $x_{ik}$ is the autoscaled value of metric *k* for amino acid *i*. These values were assembled into a distance matrix $D\in R^{n\times n}$, where each entry *d_ij_* reflects the pairwise complexity distance between amino acids *i* and *j*.

### Distance Matrices Comparison

Pearson correlation (*r*) was used to measure the linear relationship between distance values in a given pair of matrices, and was computed using SciPy's pearsonr() function. Spearman correlation (*ρ*) was used to assess rank similarity, reflecting whether amino acid distances were ordered similarly across matrices, and was computed using SciPy's spearmanr() function. To assess differences in the distribution of distances, we applied the two-sample Kolmogorov-Smirnov (KS) test, which measures the maximum difference between the empirical cumulative distribution functions (ECDFs) of two datasets. The KS test was computed using SciPy's ks_2samp() function. Distance pairs were excluded if either matrix in the comparison contained a missing value at the same position, resulting in the removal of 4 out of 190 possible amino acid pairs.

### Fraction of Single-point Mutations

The standard genetic code was used to identify all synonymous and non-synonymous single-nucleotide substitutions between amino acids. Each amino acid *a_i_* is encoded by a set of codons $C_{a_{i}}=\{c_{1},\;c_{2},\;\ldots \;,\;c_{n_{i}}\}$, where *n_i_* is the number of codons that encode *a_i_* in the genetic code. Each codon *c* = (*n_1_*, *n_2_*, *n_3_*) consists of three nucleotide positions, with *n_j_* ∈ {*A*, *U*, *G*, *C*} for *j* = 1, 2, 3. Since each nucleotide can mutate into three alternative bases, the total number of possible single-point mutations for *a_i_* is given by *M_i_*  *=*  *9·n_i_*. For an amino acid pair (*a_i_*, *a_j_*), the number of single-point mutations connecting them is: $S_{i\to j}=\sum_{c\in C_{i}}^{\;}\;\sum_{c\in C_{j}}^{\;}\;\delta (c,\;c^{\prime })$, where *δ*(*c*, *c*’) = 1 if c and c’ differ by exactly one nucleotide and 0 otherwise. The fraction of single-point mutations from *a_i_* to *a_j_* is defined as $F_{i\to j}=\frac{S_{i\to j}}{M_{i}}\;,$ which represents the probability that a random single-point mutation in a codon of *a_i_* results in *a_j_*. Position-specific mutation fractions $F_{i\to j}^{\left(p\right)}$, for codon positions *P* = {1, 2, 3}, were computed by restricting substitutions to a single-nucleotide site, as $F_{i\to j}^{\left(p\right)}=\frac{S_{i\to j}^{\left(p\right)}}{M_{i}^{\left(p\right)}}$ where $S_{i\to j}^{\left(p\right)}$ counts only single-point mutations occurring at position *P*, and $M_{i}^{\left(p\right)}=3\cdot n_{i}$ (Appendix, Supporting Methods S1). Since our goal was to assess whether high mutation fractions correspond to small differences in molecular complexity (i.e. short distances in the complexity space), we excluded amino acid pairs that had no possible single-point mutations connecting their codons.

### MDS (Euclidean Space Embedding)

Classical MDS (Kruskal 1964) was used to embed the 16-dimensional amino acid point set into a lower-dimensional Euclidean space while maximizing the accuracy of reproduced pairwise distances. The reconstructed distance matrix was obtained from the Gram matrix of vector inner products, which was approximated by truncating its spectral decomposition via singular value decomposition (SVD) and forcing the associated matrix rank to a prescribed threshold. The forced rank corresponds to actual embedding dimension and the number of retained dimensions was minimally chosen to guarantee reconstruction error of less than 2% on average over all distance pairs. Embedding into 7, 5, and 6 dimensions provided sufficient accuracy for the full set of 16 metrics, the subset of 7 graph-based metrics, and the subset of 9 information-based metrics, respectively (Appendix, Supporting Methods S2). This dimensionality reduction implicitly reflects the correlation structure among the metrics, since only shared variance allows the 16-dimensional space to be reconstructed in 6 to 7 dimensions with minimal distortion. Although the two constructs are distinct, Euclidean-metric MDS is known to be mathematically equivalent to Principal Component Analysis (PCA) which reduces the data via uncorrelated components in the latent space.

### Simplicial Complex

A VR simplicial complex (Vietoris 1927; Hausmann 1996) was constructed to encode the topological relationships among amino acids in the embedded space. A simplicial complex is a collection of simplices where each face of a simplex belongs to the complex and any intersection between simplices is a shared face. In this construction, a *k*-simplex is included if all pairwise distances among its k+1 vertices are below a threshold ϵ. The complex at scale ϵ is denoted VR(χ,ϵ) , where χ is the set of embedded amino acids and $d_{ij}$ is the Euclidean distance between all pairs χ. To ensure that all 20 amino acids formed a single connected component, we incrementally increased ε and identified the lowest threshold at which full connectivity was achieved. This value, ϵ=1.0 standard deviations per dimension (equivalent to a total distance of 7 in the 7-dimensional embedding), was then used to define the final complex (Appendix, Supporting Methods S2 and Fig. S12). The resulting connectivity graph served as the basis for MST computation.

### Minimum Spanning Tree

The MST was computed from the connectivity graph obtained at the final filtration level ϵ=1.0, where all 20 amino acids form a single connected component. The MST identifies the shortest undirected path linking all nodes admitting pre-computed pairwise complexity distances. We used Prim's algorithm (Prim 1957), a greedy method that incrementally grows the tree by adding the smallest-weight edge connecting a new node to the existing structure. Formally, given a weighted, connected graph G=(V,E), with edge weights $d_{ij}$, the MST is the spanning subset T⊆E that minimizes the total edge weight: $T=argmin_{T}\;\sum_{(i,j)\in T^{\prime }}d_{ij}$, subject to $T^{\prime }$ spans *V* (Appendix, Supporting Methods S2 and Fig. S12). Since all pairwise distances are distinct, the MST is guaranteed to be unique.

### Pairwise Tree Comparisons

Graph Edit Distance (GED) was approximated using NetworkX's optimize_graph_edit_distance function, measuring the minimum number of edge modifications needed to transform one tree into another. Adjacency Matrix Similarity (AMS) was determined by computing the normalized Hamming distance between adjacency matrices of the compared graphs. Jaccard Similarity (JAC) was calculated as the ratio of shared edges to the total number of unique edges across both trees.

### Mutational Connectivity in Randomized Trees

The number of single-point mutations *S_i_*→*_j_* connecting adjacent amino acids (*a_i_*, *a_j_*) in the MST was used to assess mutational connectivity. For each edge, we computed the fraction *F_i_*→*_j_*  *=*  *S_i_*→*_j_/M_i_*, where *M_i_* is the total number of possible single-nucleotide substitutions for *a_i_* (Appendix, Supporting Methods S1). To evaluate whether the observed MST preserved more mutational paths than expected by chance, we performed *N* = 10^5^ randomizations in which amino acids were randomly reassigned to nodes while maintaining the original MST topology. For each randomized tree, we calculated the total number of connecting mutations: $S_{total}=\sum_{(a_{i},a_{j})\in MST}^{\;}\;S_{i\to j}$, and the overall mutation fraction: $F_{total}=\sum_{(a_{i},a_{j})\in MST}^{\;}\;\frac{S_{i\to j}}{M_{i}}$ . These values were computed separately for the first, second, and third codon positions, as well as for all positions combined. For each metric, we estimated the mean *μ* and standard deviation *σ* across the randomized ensemble and defined 95% confidence intervals as *μ* ± *1.96·σ/√Ν*. The observed MST was considered significantly enriched for mutational connectivity if it exceeded the upper bound of the corresponding confidence interval.

### Mutational Connectivity in a Path Graph

As a null model for comparison, we computed the expected number of mutational connections in a randomly ordered linear sequence of amino acids modeled as a path graph $P_{20}$, a tree of 20 nodes connected by 19 unbranched edges. Each amino acid is linked to two neighbors (except the endpoints), forming 19 transitions. The background mutation rate for each codon position p∈{1,2,3} was estimated as: $F(p)=\frac{1}{N}\sum_{k=1}^{N}\;F_{k}(p)$, where $F_{k}(p)$ is the fraction of single-point mutations at position *P* that convert a codon for amino acid *a_k_* into a codon for a different amino acid, and *N* = 20 is the number of proteinogenic amino acids. This yielded values of F(1)=0.44, F(2)=0.46, and F(3)=0.13. The expected number of single-point mutations connecting consecutive amino acids in the path graph was then calculated as E(p)=(N−1)⋅F(p). Substituting the background values, we obtained E(1)=19×0.44=8.32, E(2)=19×0.46=8.59 and E(3)=19×0.13=2.89.

### Method Rank-based Approach

Each amino acid was ranked from lowest to highest complexity for each of the 16 metrics (Appendix, Table S3). Ties were resolved using a threshold of 0.005, grouping amino acids with near-identical values under the same rank. Final ranks were averaged across all metrics to produce a composite complexity score for each amino acid (Appendix, Tables S5 and S6).

## Supplementary Material

evag012_Supplementary_Data

## Data Availability

The code used to compute molecular complexity metrics and perform the subsequent analyses is available at https://github.com/celiablanco/MolecularComplexityAA.

## References

[evag012-B1] Ali T, Borah C. Analysis of amino acids network based on mutation and base positions. Gene Rep. 2021:24:101291. 10.1016/j.genrep.2021.101291.

[evag012-B2] Allu TK, Oprea TI. Rapid evaluation of synthetic and molecular complexity for in silico chemistry. J Chem Inf Model. 2005:45:1237–1243. 10.1021/ci0501387. 16180900

[evag012-B3] Balaban AT . Highly discriminating distance-based topological Index. Chem Phys Lett. 1982:89:399–404. 10.1016/0009-2614(82)80009-2.

[evag012-B4] Barone R, Chanon M. A new and simple approach to chemical complexity. Application to the synthesis of natural products. J Chem Inf Comput Sci. 2001:41:269–272. 10.1021/ci000145p. 11277709

[evag012-B5] Bartlett GJ, Porter CT, Borkakoti N, Thornton JM. Analysis of catalytic residues in enzyme active sites. J Mol Biol. 2002:324:105–121. 10.1016/S0022-2836(02)01036-7. 12421562

[evag012-B6] Becker RS, Hong K, Hong JH. Hot hydrogen atoms reactions of interest in molecular evolution and interstellar chemistry. J Mol Evol. 1974:4:157–172. 10.1007/BF01732020. 4469275

[evag012-B7] Bender A, Hajieva P, Moosmann B. Adaptive antioxidant methionine accumulation in respiratory chain complexes explains the use of a deviant genetic code in mitochondria. Proc Natl Acad Sci U S A. 2008:105:16496–16501. 10.1073/pnas.0802779105. 18946048 PMC2575448

[evag012-B8] Berlett BS, Levine RL. Designing antioxidant peptides. Redox Rep. 2014:19:80–86. 10.1179/1351000213Y.0000000078. 24520968 PMC4130572

[evag012-B9] Bertz SH . The first general Index of molecular complexity. J Am Chem Soc. 1981:103:3599–3601. 10.1021/ja00402a071.

[evag012-B10] Biter AB, et al A method to probe protein structure from UV absorbance Spectra. Anal Biochem. 2019:587:113450. 10.1016/j.ab.2019.113450. 31550438

[evag012-B11] Blanco C, Bayas M, Yan F, Chen IA. Analysis of evolutionarily independent protein-RNA complexes yields a criterion to evaluate the relevance of prebiotic scenarios. Curr Biol. 2018:28:526–537.e5. 10.1016/j.cub.2018.01.014. 29398222

[evag012-B12] Böttcher T . An additive definition of molecular complexity. J Chem Inf Model. 2016:56:462–470. 10.1021/acs.jcim.5b00723. 26857537

[evag012-B13] Böttcher T . From molecules to life: quantifying the complexity of chemical and biological systems in the universe. J Mol Evol. 2018:86:1–10. 10.1007/s00239-017-9824-6. 29260254 PMC5794832

[evag012-B14] Cleaves HJ II . The origin of the biologically coded amino acids. J Theor Biol. 2010:263:490–498. 10.1016/j.jtbi.2009.12.014. 20034500

[evag012-B15] Clemons PA, et al Small molecules of different origins have distinct distributions of structural complexity that correlate with protein-binding profiles. Proc Natl Acad Sci U S A. 2010:107:18787–18792. 10.1073/pnas.1012741107. 20956335 PMC2973913

[evag012-B16] Corominas-Murtra B, Seoane LF, Solé R. Zipf's law, unbounded complexity and open-ended evolution. J R Soc Interface. 2018:15:20180395. 10.1098/rsif.2018.0395. 30958235 PMC6303796

[evag012-B17] Crick FH . Codon—anticodon pairing: the wobble hypothesis. J Mol Biol. 1966:19:548–555. 10.1016/S0022-2836(66)80022-0. 5969078

[evag012-B18] Da Pieve F . Physicochemical properties and complexity of amino acids beyond our biosphere: analysis of the isoleucine group from meteorites. ACS Earth Space Chem. 2019:3:1955–1965. 10.1021/acsearthspacechem.9b00131.

[evag012-B19] Day T . Computability, Gödel's incompleteness theorem, and an inherent limit on the predictability of evolution. J R Soc Interface. 2012:9:624–639. 10.1098/rsif.2011.0479. 21849390 PMC3284140

[evag012-B20] Di Giulio M . An extension of the coevolution theory of the origin of the genetic code. Biol Direct. 2008:3:37. 10.1186/1745-6150-3-37. 18775066 PMC2538516

[evag012-B21] Dufton MJ . Genetic code synonym quotas and amino acid complexity: cutting the cost of proteins? J Theor Biol. 1997:187:165–173. 10.1006/jtbi.1997.0443. 9237887

[evag012-B22] Eilers M, Patel AB, Liu W, Smith SO. Comparison of Helix interactions in membrane and soluble alpha-bundle proteins. Biophys J. 2002:82:2720–2736. 10.1016/S0006-3495(02)75613-0. 11964258 PMC1302060

[evag012-B23] Fried SD, Fujishima K, Makarov M, Cherepashuk I, Hlouchova K. Peptides before and during the nucleotide world: an origins story emphasizing cooperation between proteins and nucleic acids. J R Soc Interface. 2022:19:20210641. 10.1098/rsif.2021.0641. 35135297 PMC8833103

[evag012-B24] GitHub . “GitHub—AstraZeneca/molecular-complexity: Python implementation of the molecular complexity metric described by proudfoot 2017 (10.1016/j.bmcl.2017.03.008).” Accessed January 19, 2025. https://github.com/AstraZeneca/molecular-complexity. n.d.

[evag012-B25] Gould SJ . Full house. Harvard University Press; 2011.

[evag012-B26] Gutman I, Rücker C, Rücker G. On walks in molecular graphs. J Chem Inf Comput Sci. 2001:41:739–745. 10.1021/ci000149u. 11410054

[evag012-B27] Gutman I, Ruščić B, Trinajstić N, Wilcox CF Jr. Graph theory and molecular orbitals. XII. Acyclic polyenes. J Chem Phys. 1975:62:3399–3405. 10.1063/1.430994.

[evag012-B28] Haig D, Hurst LD. A quantitative measure of error minimization in the genetic code. J Mol Evol. 1999:49:708. 10.1007/PL00006591. 10552053

[evag012-B29] Hall LH, Kier LB. The molecular connectivity chi indexes and kappa shape indexes in structure-property modeling. In: Reviews in computational chemistry. Reviews in computational chemistry. John Wiley & Sons; 2007. p. 367–422.

[evag012-B30] Hausmann J-C . On the Vietoris-rips complexes and a cohomology theory for metric spaces. In: Quinn F, editor. Prospects in topology (AM-138). Princeton University Press; 1996. p. 175–188.

[evag012-B31] Hayes B . The invention of the genetic code. Am Sci. 1998:86:8. 10.1511/1998.17.8.

[evag012-B32] Hendrickson JB, Huang P, Toczko AG. Molecular complexity: a simplified formula adapted to individual atoms. J Chem Inf Comput Sci. 1987:27:63–67. 10.1021/ci00054a004.

[evag012-B33] Hennet RJ, Holm NG, Engel MH. Abiotic synthesis of amino acids under hydrothermal conditions and the origin of life: a perpetual phenomenon? Naturwissenschaften. 1992:79:361–365. 10.1007/BF01140180. 1522920

[evag012-B34] Higgs PG . A four-column theory for the origin of the genetic code: tracing the evolutionary pathways that gave rise to an optimized code. Biol Direct. 2009:4:16. 10.1186/1745-6150-4-16. 19393096 PMC2689856

[evag012-B35] Higgs PG, Pudritz RE. A thermodynamic basis for prebiotic amino acid synthesis and the nature of the first genetic code. Astrobiology. 2009:9:483–490. 10.1089/ast.2008.0280. 19566427

[evag012-B36] Jirasek M, et al Investigating and quantifying molecular complexity using assembly theory and spectroscopy. ACS Cent Sci. 2024:10:1054–1064. 10.1021/acscentsci.4c00120. 38799656 PMC11117308

[evag012-B37] Jones S, Daley DT, Luscombe NM, Berman HM, Thornton JM. Protein-RNA interactions: a structural analysis. Nucleic Acids Res. 2001:29:943–954. 10.1093/nar/29.4.943. 11160927 PMC29619

[evag012-B38] Jukes TH . Possibilities for the evolution of the genetic code from a preceding form. Nature. 1973:246:22–26. 10.1038/246022a0. 4585842

[evag012-B39] Kaiser F, et al The structural basis of the genetic code: amino acid recognition by aminoacyl-tRNA synthetases. Sci Rep. 2020:10:14647. 10.1038/s41598-020-69100-0. 32724042 PMC7387524

[evag012-B40] Kalambokidis M, Travisano M. The eco-evolutionary origins of life. Evolution. 2024:78:1–12. 10.1093/evolut/qpad195. 37930681

[evag012-B41] Kim OTP, Yura K, Go N. Amino acid residue doublet propensity in the protein-RNA interface and its application to RNA interface prediction. Nucleic Acids Res. 2006:34:6450–6460. 10.1093/nar/gkl819. 17130160 PMC1761430

[evag012-B42] Kim S, et al PubChem 2023 update. Nucleic Acids Res. 2023:51:D1373–D1380. 10.1093/nar/gkac956. 36305812 PMC9825602

[evag012-B43] Koonin EV, Novozhilov AS. Origin and evolution of the genetic code: the universal Enigma. IUBMB Life. 2009:61:99–111. 10.1002/iub.146. 19117371 PMC3293468

[evag012-B44] Kruskal JB . Multidimensional scaling by optimizing goodness of fit to a nonmetric hypothesis. Psychometrika. 1964:29:1–27. 10.1007/BF02289565.

[evag012-B45] Krzyzanowski A, Pahl A, Grigalunas M, Waldmann H. Spacial score─A comprehensive topological indicator for small-molecule complexity. J Med Chem. 2023:66:12739–12750. 10.1021/acs.jmedchem.3c00689. 37651653 PMC10544027

[evag012-B46] Landrum G, et al 2023, February 23. Rdkit/rdkit: 2022_09_5 (Q3 2022) Release. Version 2022_09_5. [Computer software]. Zenodo. 10.5281/zenodo.7671152.

[evag012-B47] Li J, Eastgate MD. Current complexity: a tool for assessing the complexity of organic molecules. Org Biomol Chem. 2015:13:7164–7176. 10.1039/C5OB00709G. 25962620

[evag012-B48] Liu X, et al Genome wide exploration of the origin and evolution of amino acids. BMC Evol Biol. 2010:10:77. 10.1186/1471-2148-10-77. 20230639 PMC2853539

[evag012-B49] Longo LM, Blaber M. Protein design at the interface of the Pre-biotic and biotic worlds. Arch Biochem Biophys. 2012:526:16–21. 10.1016/j.abb.2012.06.009. 22772066

[evag012-B50] Longo LM, Lee J, Blaber M. Simplified protein design biased for prebiotic amino acids yields a foldable, halophilic protein. Proc Natl Acad Sci U S A. 2013:110:2135–2139. 10.1073/pnas.1219530110. 23341608 PMC3568330

[evag012-B51] Lovering F, Bikker J, Humblet C. Escape from Flatland: increasing Saturation as an Approach to Improving Clinical Success. J Med Chem. 2009:52:6752–6756. 10.1021/jm901241e. 19827778

[evag012-B52] Mayer C . Life in the context of order and complexity. Life. 2020:10:5. 10.3390/life10010005. 31963637 PMC7175320

[evag012-B53] McDonald GD, Storrie-Lombardi MC. Biochemical constraints in a protobiotic earth devoid of basic amino acids: the “BAA(-) world”. Astrobiology. 2010:10:989–1000. 10.1089/ast.2010.0484. 21162678

[evag012-B54] McShea DW, Brandon RN. Biology's first law: The tendency for diversity and complexity to increase in evolutionary systems. University of Chicago Press; 2010.

[evag012-B55] Moriwaki H, Tian Y-S, Kawashita N, Takagi T. Mordred: a molecular descriptor calculator. J Cheminform. 2018:10:4. 10.1186/s13321-018-0258-y. 29411163 PMC5801138

[evag012-B56] Moya A . The calculus of life. Towards a theory of life. Springer; 2015.

[evag012-B57] Nikolic S, Tolic I, Trinajstic N, Baucic I. On the Zagreb indices as complexity indices. Croat Chem Acta. 2000:73:909–921.

[evag012-B58] Nikolić S, Trinajstić N, Tolić IM, Rucker G, Rucker C. On molecular complexity indices. In: Bonchev D, Rouvray DH, editors. Complexity in chemistry. Introduction and fundamentals. CRC Press; 2003. p. 23–76.

[evag012-B59] Nören-Müller A, et al Discovery of protein phosphatase inhibitor classes by biology-oriented synthesis. Proc Natl Acad Sci U S A. 2006:103:10606–10611. 10.1073/pnas.0601490103. 16809424 PMC1502279

[evag012-B60] Novozhilov AS, Wolf YI, Koonin EV. Evolution of the genetic code: partial optimization of a random code for robustness to translation error in a rugged fitness landscape. Biol Direct. 2007:2:24. 10.1186/1745-6150-2-24. 17956616 PMC2211284

[evag012-B61] Nuevo M, Auger G, Blanot D, d’Hendecourt L. A detailed study of the amino acids produced from the vacuum UV irradiation of interstellar ice analogs. Orig Life Evol Biosph. 2008:38:37–56. 10.1007/s11084-007-9117-y. 18175206

[evag012-B62] Ogle JM, et al Recognition of cognate transfer RNA by the 30*S* ribosomal subunit. Science. 2001:292:897–902. 10.1126/science.1060612. 11340196

[evag012-B63] Ogle JM, Carter AP, Ramakrishnan V. Insights into the decoding mechanism from recent ribosome structures. Trends Biochem Sci. 2003:28:259–266. 10.1016/S0968-0004(03)00066-5. 12765838

[evag012-B64] Ogle JM, Ramakrishnan V. Structural insights into translational fidelity. Annu Rev Biochem. 2005:74:129–177. 10.1146/annurev.biochem.74.061903.155440. 15952884

[evag012-B65] Oprea TI, Bologa C. Molecular complexity: you know it when you see it. J Med Chem. 2023:66:12710–12714. 10.1021/acs.jmedchem.3c01507. 37675804 PMC10544322

[evag012-B66] Papentin F . On order and complexity. II. Application to chemical and biochemical structures. J Theor Biol. 1982:95:225–245. 10.1016/0022-5193(82)90241-7. 6177972

[evag012-B67] Parker ET, et al Prebiotic synthesis of methionine and other Sulfur-containing organic compounds on the primitive earth: a contemporary reassessment based on an unpublished 1958 stanley miller experiment. Orig Life Evol Biosph. 2011:41:201–212. 10.1007/s11084-010-9228-8. 21063908 PMC3094541

[evag012-B68] Prim RC . Shortest connection networks and some generalizations. Bell Syst Tech J. 1957:36:1389–1401. 10.1002/j.1538-7305.1957.tb01515.x.

[evag012-B69] Proudfoot JR . A path based approach to assessing molecular complexity. Bioorg Med Chem Lett. 2017:27:2014–2017. 10.1016/j.bmcl.2017.03.008. 28325603

[evag012-B70] Raggi L, Bada JL, Lazcano A. On the lack of evolutionary continuity between prebiotic peptides and extant enzymes. Phys Chem Chem Phys. 2016:18:20028–20032. 10.1039/C6CP00793G. 27121024

[evag012-B71] Rozhoňová H, Martí-Gómez C, McCandlish DM, Payne JL. Robust genetic codes enhance protein evolvability. PLoS Biol. 2024:22:e3002594. 10.1371/journal.pbio.3002594. 38754362 PMC11098591

[evag012-B72] Ruecker G, Ruecker C. Counts of all walks as atomic and molecular descriptors. J Chem Inf Comput Sci. 1993:33:683–695. 10.1021/ci00015a005.

[evag012-B73] Schindeldecker M, Moosmann B. Protein-borne methionine residues as structural antioxidants in mitochondria. Amino Acids. 2015:47:1421–1432. 10.1007/s00726-015-1955-8. 25859649

[evag012-B74] Schuffenhauer A, Brown N, Selzer P, Ertl P, Jacoby E. Relationships between molecular complexity, biological activity, and structural diversity. J Chem Inf Model. 2006:46:525–535. 10.1021/ci0503558. 16562980

[evag012-B75] Sharma A, et al 2023, June 8. Assembly Theory Explains and Quantifies Selection and Evolution. Version v1. [Computer software]. Zenodo. 10.5281/ZENODO.8017327. PMC10567559 37794189

[evag012-B76] Sheridan RP, et al Modeling a crowdsourced definition of molecular complexity. J Chem Inf Model. 2014:54:1604–1616. 10.1021/ci5001778. 24802889

[evag012-B77] Spitzer J, Pielak GJ, Poolman B. Emergence of life: physical chemistry changes the paradigm. Biol Direct. 2015:10:33. 10.1186/s13062-015-0060-y. 26059688 PMC4460864

[evag012-B78] Tenaillon O, Matic I. The impact of neutral mutations on genome evolvability. Curr Biol. 2020:30:R527–R534. 10.1016/j.cub.2020.03.056. 32428494

[evag012-B79] Treger M, Westhof E. Statistical analysis of atomic contacts at RNA-protein interfaces. J Mol Recognit. 2001:14:199–214. 10.1002/jmr.534. 11500966

[evag012-B80] Trifonov EN . Consensus temporal order of amino acids and evolution of the triplet code. Gene. 2000:261:139–151. 10.1016/S0378-1119(00)00476-5. 11164045

[evag012-B81] Trifonov EN . The triplet code from first principles. J Biomol Struct Dyn. 2004:22:1–11. 10.1080/07391102.2004.10506975. 15214800

[evag012-B82] UniProt Consortium . UniProt: the universal protein knowledgebase in 2025. Nucleic Acids Res. 2025:53:D609–D617. 10.1093/nar/gkae1010. 39552041 PMC11701636

[evag012-B83] Van Trump JE, Miller SL. Prebiotic synthesis of methionine. Science. 1972:178:859–860. 10.1126/science.178.4063.859. 5085982

[evag012-B84] Vietoris L . Über den höheren zusammenhang kompakter räume und eine klasse von zusammenhangstreuen abbildungen. Math Ann. 1927:97:454–472. 10.1007/BF01447877.

[evag012-B85] von Korff M, Sander T. Molecular complexity calculated by fractal dimension. Sci Rep. 2019:9:967. 10.1038/s41598-018-37253-8. 30700728 PMC6353876

[evag012-B86] Wehbi S, et al Order of amino acid recruitment into the genetic code resolved by last universal common Ancestor's protein domains. Proc Natl Acad Sci U S A. 2024:121:e2410311121. 10.1073/pnas.2410311121. 39665745 PMC11670089

[evag012-B87] Whitlock HW . On the structure of total synthesis of Complex natural products. J Org Chem. 1998:63:7982–7989. 10.1021/jo9814546.

[evag012-B88] Wong JT . A co-evolution theory of the genetic code. Proc Natl Acad Sci U S A. 1975:72:1909–1912. 10.1073/pnas.72.5.1909. 1057181 PMC432657

[evag012-B89] Wright BA, et al Molecular complexity-inspired synthetic strategies toward the calyciphylline A-type *Daphniphyllum* alkaloids himalensine A and daphenylline. J Am Chem Soc. 2024:146:33130–33148. 10.1021/jacs.4c11252. 39565045 PMC12430491

[evag012-B90] Wright BA, Sarpong R. Molecular complexity as a driving force for the advancement of organic synthesis. Nat Rev Chem. 2024:8:776–792. 10.1038/s41570-024-00645-8. 39251714 PMC11608557

[evag012-B91] Zhao M, Ding R, Liu Y, Ji Z, Zhao Y. Determination of the amino acid recruitment order in early life by genome-wide analysis of amino acid usage bias. Biomolecules. 2022:12:171. 10.3390/biom12020171. 35204672 PMC8961565

