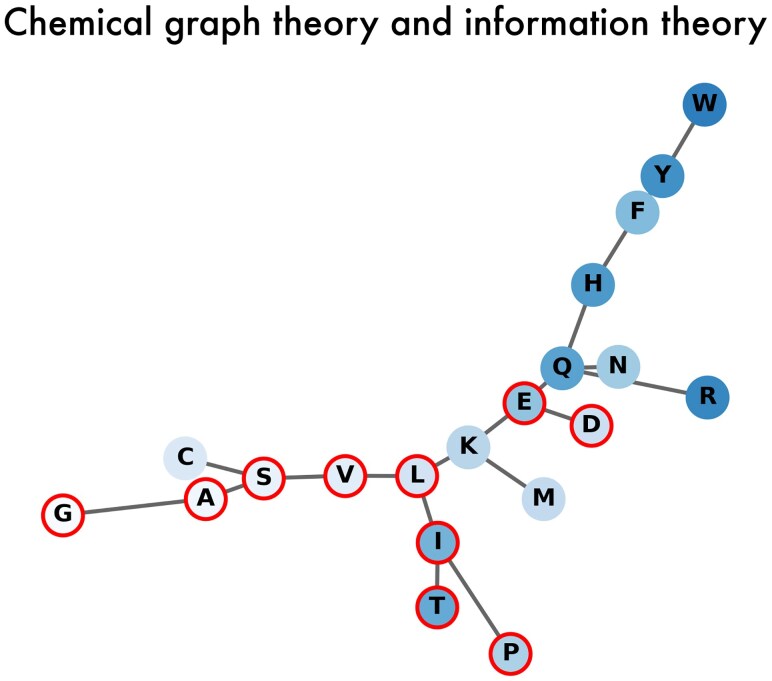# Correction to: Molecular Complexity Constrained Early Amino Acid Recruitment into the Genetic Code

**DOI:** 10.1093/gbe/evag143

**Published:** 2026-06-17

This is a correction to: Syeda Ameena Hashmi, Hamed Chok, Ricardo Cabrera, Celia Blanco, Molecular Complexity Constrained Early Amino Acid Recruitment into the Genetic Code, *Genome Biology and Evolution*, Volume 18, Issue 3, March 2026, evag012, https://doi.org/10.1093/gbe/evag012

In the originally published version of the paper, in Figure 4, the lower part of panel (b) was lacking. The image is now corrected so that all sections are showing.

**Figure:**