# Supplementary material for: Molecular Complexity Constrained Early Amino Acid Recruitment into the Genetic Code
Source: Genome Biol Evol. 2026 Jan 20;18(3):evag012. doi: 10.1093/gbe/evag012 (PMC12951668; doi:10.1093/gbe/evag012)
Supplement: evag012_Supplementary_Data [file evag012_supplementary_data.pdf]

Supporting Information for

Molecular Complexity Constrained Early Amino Acid Recruitment into the Genetic code

Syeda Ameena Hashmi[a,1], Hamed Chok[a,1], Ricardo Cabrera[b], Celia Blanco[a,2]

1. These authors contributed equally
2. Corresponding author: celiablanco@bmsis.org

This PDF file includes:

Supporting Methods S1 to S2
Figures S1 to S14
Tables S1 to S6
SI References

Other supporting materials for this manuscript include the following:

Code used to compute molecular complexity metrics and perform the associated analyses is available at https://github.com/celiablanco/MolecularComplexityAA

Supporting Methods S1. Calculation of fraction of single-point mutations.

To illustrate this computation, consider the transition between glycine (G) and alanine (A). Glycine is encoded by the codons C_G = {GGU, GGC, GGA, GGG}, while alanine is encoded by C_A = {GCU, GCC, GCA, GCG}. A single-point mutation can convert GGU (glycine) to GCU (alanine) by mutating the second nucleotide from G to C. Similarly, GGC can mutate to GCC, GGA to GCA, and GGG to GCG by a single change at the second position. In this case, the total number of mutations $S_{G\rightarrow A}$ is 4, as each glycine codon has exactly one corresponding alanine codon differing by a single substitution. Since glycine has n_G = 4 codons, the total number of possible single-point mutations is M_G = 9 · 4 = 36. The fraction of single-point mutations leading from glycine to alanine is therefore $F_{G\rightarrow A}$ = 4/36 = 0.111.

This fraction can be further broken down by position-specific mutations. In this example, all transitions occur at the second codon position, meaning $S^{(2)}_{G\rightarrow A} = 4$, while $S^{(1)}_{G\rightarrow A} = S^{(3)}_{G\rightarrow A} = 0$, leading to $F^{(1)}_{G\rightarrow A} = 0$, $F^{(2)}_{G\rightarrow A} = 4/12 = 0.333$, $S^{(2)}_{G\rightarrow A} = 0$, where $M^{(p)}_{G} = 3 \cdot n_{G} = 12$ for each codon position.

Supporting Methods S2. Dimensionality reduction and topological analysis of amino acid complexity space.

The algorithm used to compute the final minimum spanning tree (MST) involves three main steps, preceded by a preprocessing stage.

Preprocessing. All individual metrics were autoscaled (mean-centered with unit standard deviation) to eliminate differences in scale and ensure uniform contribution to the Euclidean distance calculations.

Step 1: Multidimensional scaling (Euclidean space embedding). This step reconstructs the original pairwise distances in the raw feature space $\mathbb{R}^p$ (with $p = 16$ dimensions) by embedding them into a lower-dimensional Euclidean space $\mathbb{R}^d$, with $d < p$. The goal is to improve the efficiency of both data representation and subsequent computation while recovering inherent data dimensionality. To accomplish this, we used the classical multidimensional scaling (MDS) algorithm [1]. The method relies on the fact that the matrix of squared Euclidean distances $D \in \mathbb{R}^{n\times n}$, where $D_{ij} = ||x_i - x_j||^2$, is linearly related to the associated Gram matrix $G \in \mathbb{R}^{n\times n}$ [2], where $G_{ij} = \langle x_i, x_j \rangle$, containing the inner products between all possible vector pairs. The underlying linear Gram-to-squared-distance-matrix mapping may be written as: $D_{ij} = D_{i0} + D_{j0} - 2G_{ij}$ where D_{i0} and D_{j0} denote the squared distances to the origin from vectors x_i and x_j , respectively.

Furthermore, the rank of the Gram matrix determines the required number of dimensions needed to losslessly reproduce all squared Euclidean distances. Hence, while G may be full rank (i.e., rank 16), it is appropriate to seek a lower-rank approximation $\widetilde{G}$ that yields a lower-dimensional embedding space.

The optimal approximating Gram matrix $\widetilde{G}$ minimizes the Frobenius norm of the difference from the original: $\widetilde{G} = argmin_{G'} ||G - G'||_F$, and is obtained by setting the lowest eigenvalues of G to zero. For example, to produce a 10-dimensional embedding, the six smallest eigenvalues are discarded. The

eigenvalue decomposition was performed using singular value decomposition (SVD), a standard matrix factorization technique: $G = U\Lambda U^{\top}$, where Λ is the diagonal matrix of eigenvalues and U the corresponding matrix of eigenvectors (noting the special case that G is positive definite by definition in our application).

To determine the appropriate embedding dimension, we iteratively removed the lower eigenvalues until the resulting pairwise distances (recomputed from the truncated embedding) yielded an average reconstruction error below 2%. For all cases, this threshold was used to determine the minimum embedding dimension. For the full set of 16 metrics, embedding into 7 dimensions was sufficient to reproduce the original 16-dimensional distances within this threshold (SI Appendix, Fig. S6). For the subset of 7 graph-based metrics, and the subset of 9 information-based metrics, embedding into 5- and 6-dimensional spaces, respectively, also satisfied the <2% error criterion.

Step 2: Simplicial Complex. A simplex may be defined as a higher-dimensional generalization of the familiar 2D triangle structure i.e., a *d*-simplex is a set of affinely independent set of *d*+1 points. A simplicial complex is a finite set $\mathcal{K}$ of simplices (points, edges, triangles, etc.) that satisfies two conditions: (1) every face of a simplex in $\mathcal{K}$ is also in $\mathcal{K}$, and (2) the intersection of any two simplices in $\mathcal{K}$ is either empty or a common face of both. A simplicial decomposition of a point cloud, also referred to as triangulation, produces a simplicial complex over said point cloud. In abstract terms, the simplicial complex serves as a topological representation of data connectivity within (in this case) a metric space.

Our goal in constructing a simplicial complex over the embedded amino acid point cloud $\mathcal{X} \subset \mathbb{R}^{d}$ is to recover both local neighborhood structure and global connectivity among the nodes. This structure imposes constraints on valid paths through the complexity space: specifically, if two amino acids a_i and a_j are not connected by a 1-simplex (i.e., edge), then they are not considered adjacent in any path to be inferred from the complex. More generally, any sequence of amino acids must proceed via a chain of edges that form 1-faces of simplices in the complex.

While multiple constructions exist for simplicial complexes (e.g., Čech, Alpha), we used the Vietoris-Rips complex [3,4], which defines simplices on all subsets of points whose pairwise distances are less than or equal to a threshold ϵ. Formally, the Vietoris-Rips complex of $\mathcal{X}$ at scale ϵ, denoted $VR(\mathcal{X}, \epsilon)$, contains a k-simplex for every set of $k + 1$ points $\{x_0, \cdots, x_k\} \subset \mathcal{X}$ such that $||x_i - x_j|| \leq \epsilon$ for all i, j. Rather than fixing a single value of ϵ, we used a filtration approach to study the structure at multiple levels of granularity. A filtration is a nested sequence of complexes: $VR(\mathcal{X}, \epsilon_1) \subseteq VR(\mathcal{X}, \epsilon_2) \subseteq \cdots \subseteq VR(\mathcal{X}, \epsilon_m)$ for increasing values $\epsilon_1 < \epsilon_2 < \cdots < \epsilon_m$. This provides a multi-scale representation of the topological structure of $\mathscr{X}$.

We generated the filtered Vietoris-Rips complex across six normalized thresholds: $\epsilon \in \{0.2, 0.3, 0.35, 0.4, 0.6, 1.0\}$. These thresholds were defined by normalizing pairwise Euclidean distances by the number of embedding dimensions $d = 7$, such that a normalized distance ϵ corresponds to an unnormalized Euclidean distance of $\epsilon \cdot d$. For example, a threshold of $\epsilon = 1.0$ corresponds to an unnormalized Euclidean distance of 7, and $\epsilon = 0.5$ corresponds to a distance of 3.5. Since the data were autoscaled during preprocessing (i.e., each feature has unit variance), these thresholds can be interpreted as an average of ϵ standard deviations per dimension. Formally, this can be expressed as: $\epsilon = \frac{||x_i - x_j||_2}{d}, x_i, x_j \in \mathbb{R}^d$.

At $\epsilon = 0.2$ (**SI Appendix, Fig. S13A**), only six amino acids form three disconnected edge pairs: (L, K), (D, E), and (N, Q). The remaining 14 amino acids remain isolated (unpictured). At $\epsilon = 0.3$ (**SI Appendix, Fig. S13B)**, additional edges appear. Three main components are observed: (1) a new pair (F, Y); (2) the two edges (D, E) and (N, Q) now extend into two 2-simplices (triangles), forming (D, E, Q) and (D, N, Q); and (3) a linear path connecting A to K via S–V–L. At $\epsilon = 0.35$ (**SI Appendix, Fig. S13C)**, the structure consists of two connected components: (1) the pair (F, Y), and (2) a larger tree-like component of 12 amino acids. Notably, the two adjacent triangles (D, E, Q) and (D, N, Q) merge into a 3-simplex (tetrahedron) involving the four nodes (D, N, E, Q). At $\epsilon = 0.4$ (**SI Appendix, Fig. S13D)**, the same set of nodes remains connected, with additional 1- and 2-simplices increasing local density, but (F, Y) still

remains a separate component. At $\epsilon = 0.6$ (**SI Appendix, Fig. S13E)**, the number of connected amino acids increases to 18, leaving only P and R as isolated. At $\epsilon = 1.0$ (**SI Appendix, Fig. S13F)**, all 20 amino acids are connected within a single component. The resulting structure is densely interconnected, with 19 nodes forming a well-connected subgraph, and W remaining sparsely linked.

Step 3: Minimum spanning tree (MST). The filtered simplicial complex provides a multi-scale representation of connectivity among the 20 amino acids. To extract a more compact structure that preserves proximity-based relationships among amino acids, we computed the MST of the graph derived from the coarsest level of filtration that yields a fully connected graph (i.e., filtration threshold $\epsilon = 1.0$). Only the connections present in the Vietoris-Rips complex at this threshold were used as candidate edges in the MST construction. This tree provides a minimal scaffold that connects all amino acids using the shortest possible total distance based on molecular complexity. At this threshold, the Vietoris-Rips complex forms a single connected component, ensuring that the MST spans all nodes in the point cloud. Formally, given a connected, undirected graph $G = (V, E)$, where V is the set of amino acids and E the set of pairwise edges weighted by their Euclidean distances d_{ij}, the MST is a subset $T \subseteq E$ that connects all nodes in V, contains no cycles, and minimizes the total edge weight: $T = argmin_{T} \sum_{(i,j)\in T'} d_{ij}$, subject to T' spans V.

Since all pairwise distances d_{ij} are unique (for this data), the MST is guaranteed to be unique. We computed the MST using Prim's algorithm [5], a greedy iterative method that initializes from an arbitrary node and grows the tree by repeatedly adding the smallest-weight edge that connects a new node to the existing structure. The resulting tree includes 19 edges spanning all 20 amino acids, with edge weights corresponding to pairwise distances in the complexity space (**SI Appendix, Fig. S14**).

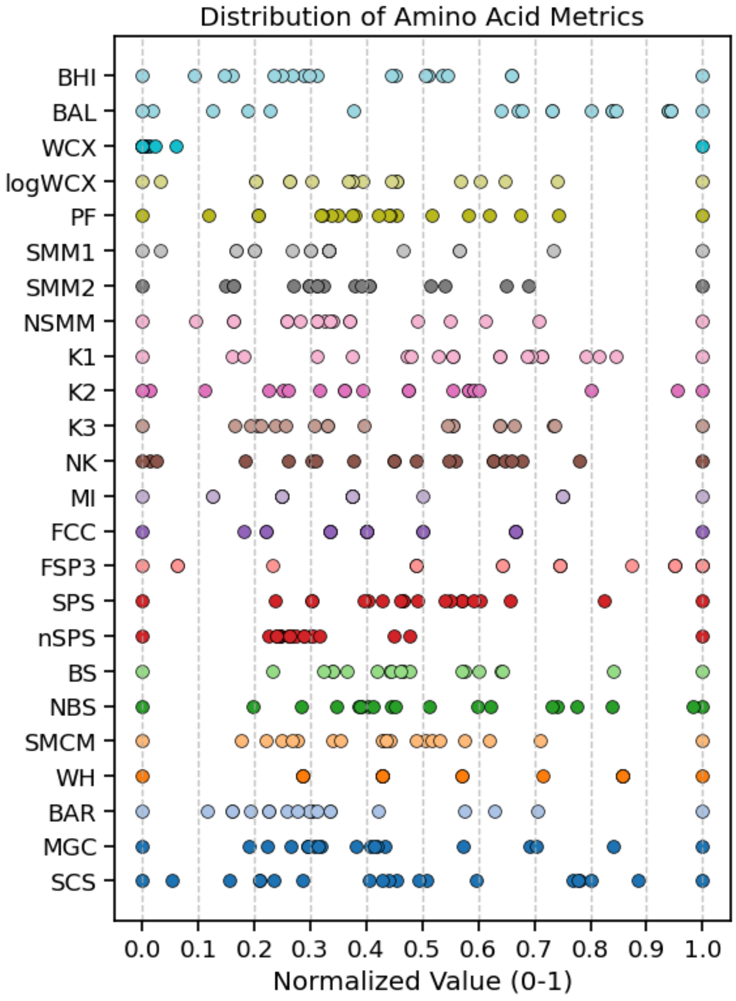

Figure S1. Normalized distribution of selected amino acid metrics and their derivates. The plot displays normalized values (scaled between 0 and 1) for 16 chosen molecular complexity metrics across 20 amino acids. Each row represents a specific metric, with individual data points indicating the relative values for each amino acid.

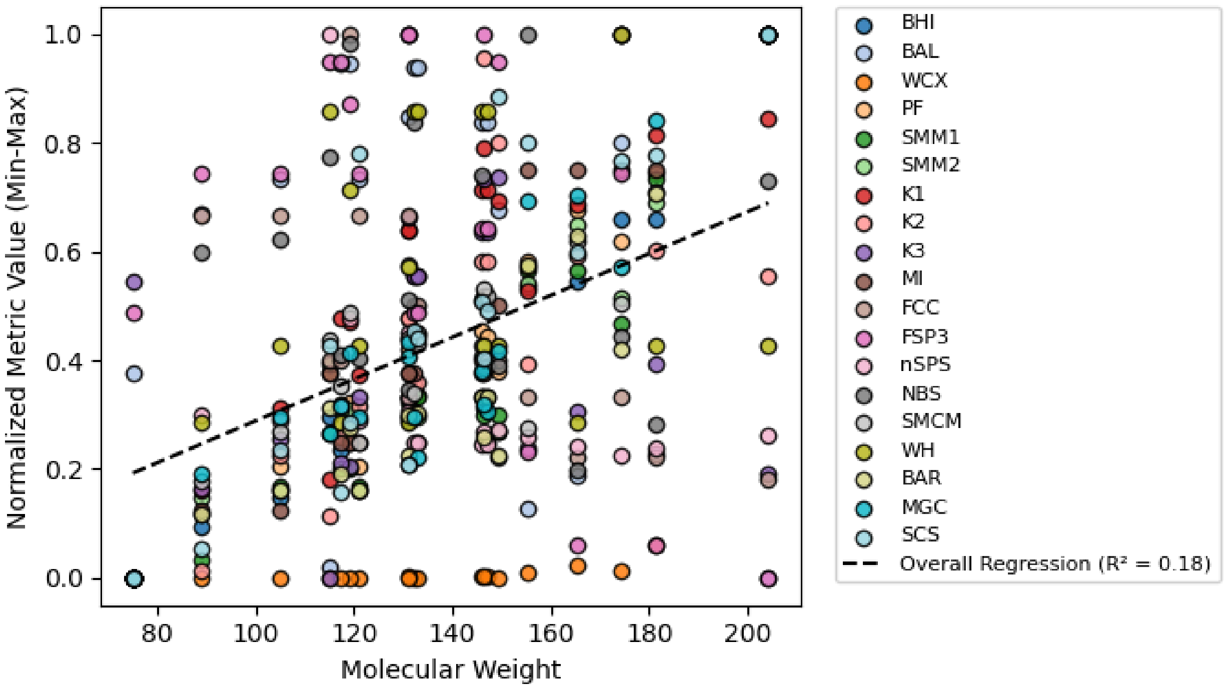

Figure S2. Relationship between molecular weight and structural complexity across the 20 proteinogenic amino acids. Each point represents the normalized value (min–max scaled) of one of the original 16 complexity metrics for a single amino acid, plotted against its molecular weight. The dashed line indicates the overall linear regression across all datapoints (N = 320), yielding a low correlation ($R^2 = 0.18$). The combination of the 16 metrics captures structural and topological features not explained by mass alone.

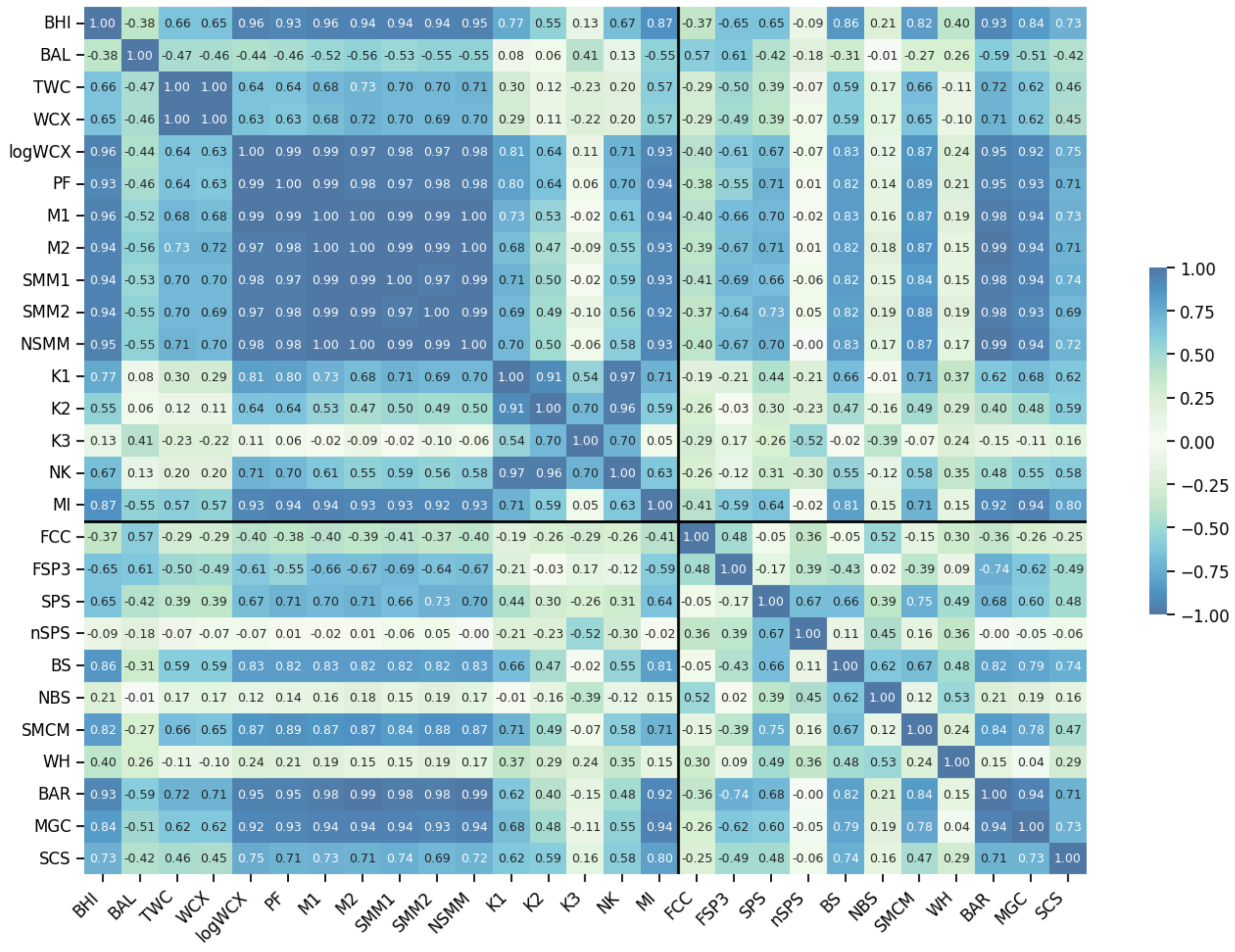

Figure S3. Symmetric matrix showing Pearson correlations among all molecular complexity metrics considered in this study, including both original and processed values. This includes untransformed and transformed metrics (WCX and logWCX), unnormalized and normalized metrics (SPS and nSPS; BS and NBS), as well as individual components and their corresponding composite norms (SMM1 and SMM2 with NSMM; K1–K3 with NK). Black, thick lines represent the division between graph theory-based metrics and information theory-based metrics.

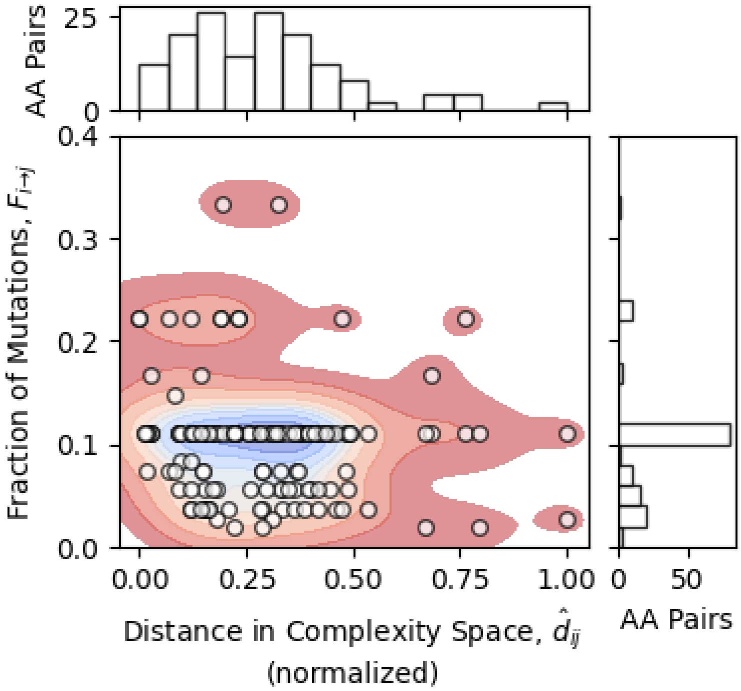

Figure S4. Relationship between normalized molecular complexity distance, $\hat{d}_{ij}$, and the fraction of possible single-point mutations ($F_{i \to j}$), for amino acids (a_i, a_j). The central scatter plot displays all amino acid pairs, with marginal histograms showing the overall distributions of normalized distances (top) and mutation fractions (right). Contour colors reflect point density based on a 2D kernel density estimate (KDE), with warmer colors indicating higher density.

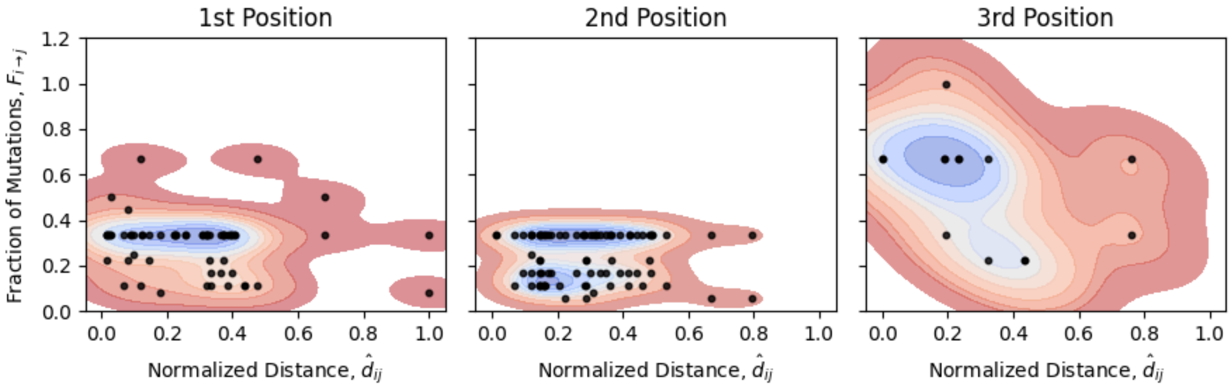

Figure S5. Relationship between normalized molecular complexity distance (d_{ij}), and the fraction of possible single-point mutations ($F_{i\to j}$), for amino acids (a_i, a_j). Black dots represent individual amino acid pairs, and contour colors indicate density from a 2D kernel density estimate (KDE), with warmer colors representing higher density. A) Combined data across all codon positions, with marginal histograms showing the distribution of distances (top) and mutation fractions (right). B) Same analysis as in A, shown separately for each codon position (1st, 2nd, and 3rd).

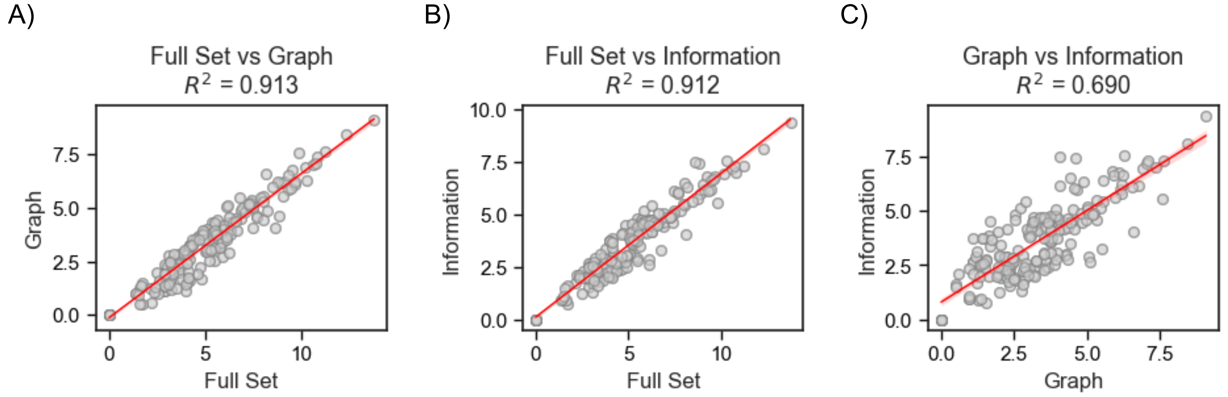

Figure S6. Pairwise Pearson correlations of amino acid distance matrices derived from the full set of 16 molecular complexity metrics, the 7 graph-theory-based metrics, and the 9 information-theory-based metrics. Each scatterplot compares distance values for 186 amino acid pairs shared across all three frameworks (4 of 190 pairs were excluded due to missing values in at least one matrix). A) Full Set vs Graph-Theory Subset ($R^2 = 0.913$), B) Full Set vs Information-Theory Subset ($R^2 = 0.912$), and C) Graph-Theory vs Information-Theory Subsets ($R^2 = 0.690$). Each plot includes a red linear regression line with a 95% confidence interval shaded in red. The high R^2 values for the comparisons involving the full set indicate strong agreement with both the graph- and information-based frameworks. The lower R^2 between the graph- and information-based frameworks reflects greater structural divergence between these two metric types. We also evaluated matrix similarity using Spearman correlation (ρ), which measures whether amino acid distances are ranked similarly across the matrices. Both frameworks correlate strongly with the full set (graph: $\rho = 0.941$; info: $\rho = 0.937$), although their correlation with each other was substantially lower ($\rho = 0.787$). The Kolmogorov-Smirnov (KS) test confirmed significant differences in their distance distributions ($p < 0.001$).

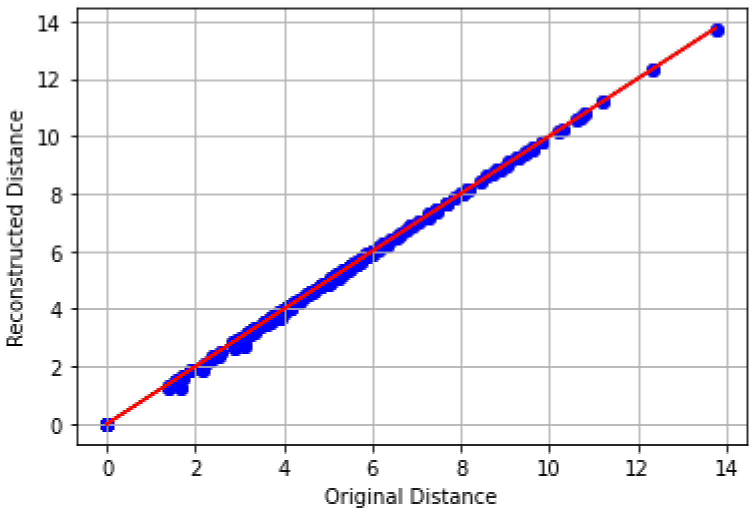

Figure S7. Comparison of original (16 dimensions) and reconstructed (7 dimensions) distances for the point cloud embedding. The high alignment along the diagonal (red line) indicates that the embedding accurately preserves the original distance structure.

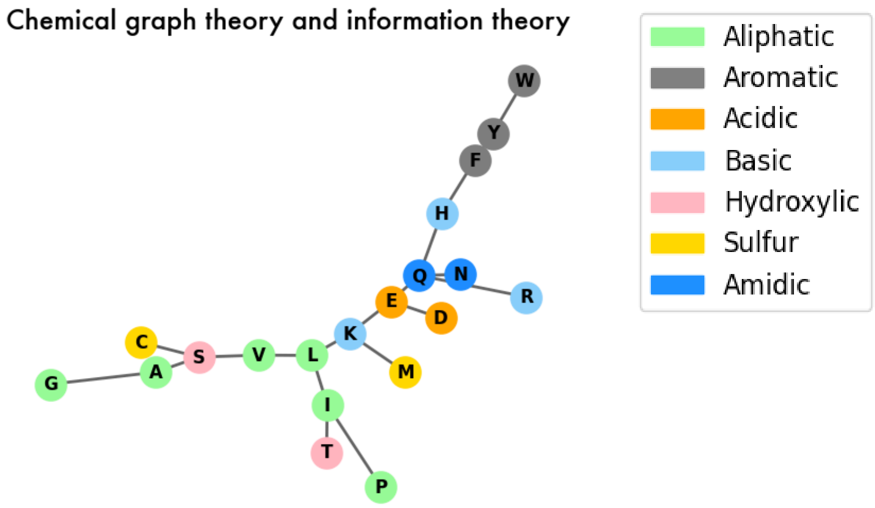

Figure S8. MST of amino acids constructed from the full set of sixteen molecular complexity metrics. Nodes are colored according to dominant physicochemical properties: aliphatic (green), aromatic (gray), acidic (orange), basic (light blue), hydroxylic (pink), sulfur-containing (yellow), and amidic (dark blue). Edge length is proportional to the pairwise distance between amino acids in the underlying complexity metric space.

A) B)

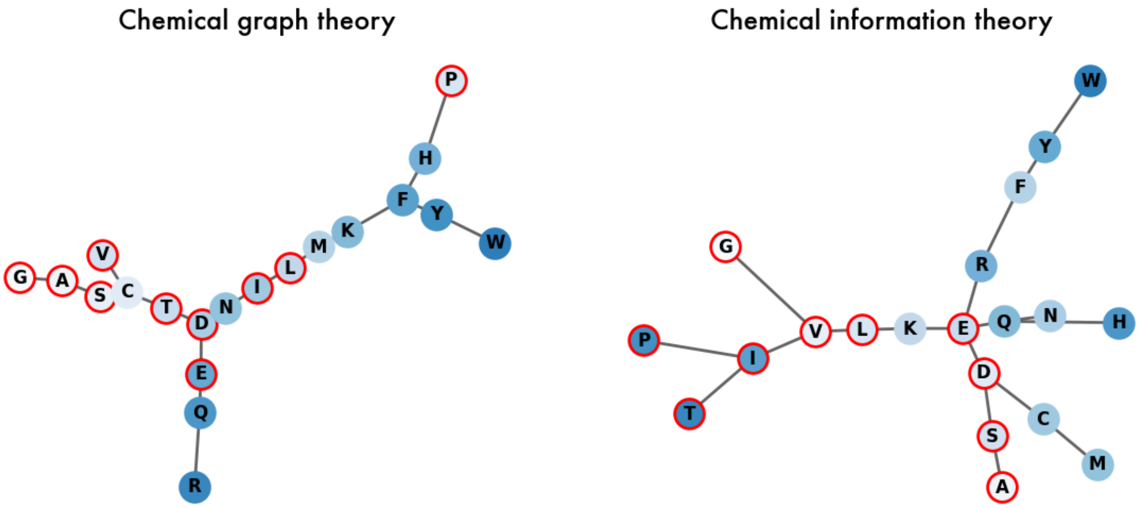

Figure S9. A) MST based on graph-theory metrics, and B) MST based on information-theory metrics. The graph-theory-based metrics were embedded into a 5-dimensional Euclidean space, and the information-theory-based metrics into a 6-dimensional space, each yielding a distance reconstruction error below 2% (SI Appendix, Supporting Methods S2). Amino acids are color-coded according to Trifonov's proposed chronology [6], with lighter colors indicating 'early' amino acids and darker colors representing 'later' ones . Nodes with thick, red edges denote amino acids supported by prebiotic evidence from meteoritic analyses, simulated prebiotic chemistry, and hydrothermal vent experiments [7]. Edges length is made proportional to weight in the tree connections; however, distances between nodes that are not directly connected do not necessarily reflect their original pairwise distances in the metric space. The tree structures were compared using three complementary metrics: Graph Edit Distance (GED), which quantifies the number of edge insertions or deletions required to transform one tree into another; Adjacency Matrix Similarity (AMS), which measures global topological similarity; and Jaccard Similarity (JAC), which reflects local edge overlap. The greatest structural difference was observed between the graph- and information-based trees (GED = 32, AMS = 0.84, JAC = 0.15). In comparison, the full-set tree was more similar to each individual framework (graph–full: GED = 28, AMS = 0.86, JAC = 0.31; info–full: GED = 30, AMS = 0.85, JAC = 0.36). For reference, the maximum possible GED between a linear and a star-like tree of N nodes is 2(N−2); in this case, GED = 36.

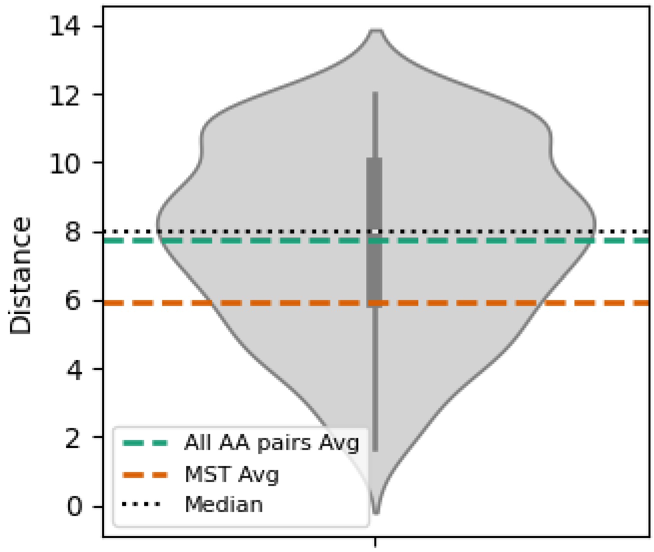

Figure S10. Distribution of pairwise amino acid distances derived from the codon-based matrix [8], which assigns distances based on the number and type of point mutations (transitions or transversions) required to convert codons of one amino acid into those of another, with position-dependent weights. The violin plot shows the full distribution of all unique amino acid pairs, with the inner boxplot marking the interquartile range and median (dotted line; median = 8). The average distance across all pairs is shown as a green dashed line (mean = 7.76), while the orange dashed line marks the average distance among amino acid pairs in our MST (mean = 5.93). The MST value lies well below both the mean and median of the full distribution, indicating that the selected connections favor mutation proximity. The distribution is skewed toward higher values because most codon comparisons between different amino acids involve mutations at the first or second codon positions and/or transversions, both of which are heavily penalized in the calculation of the distance matrix.

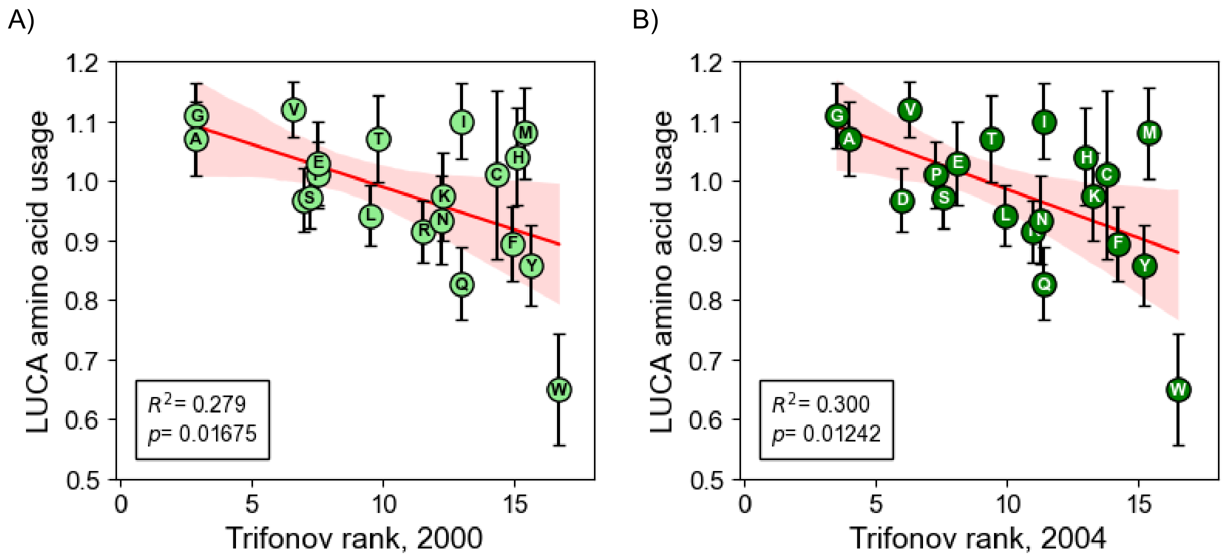

Figure S11. Correlation between LUCA amino acid usage, with error bars indicating SE [9], and A) Trifonov ranking from 2000 [10], and B) Trifonov ranking from 2004 [6,10]. Black lines represent linear regression fits.

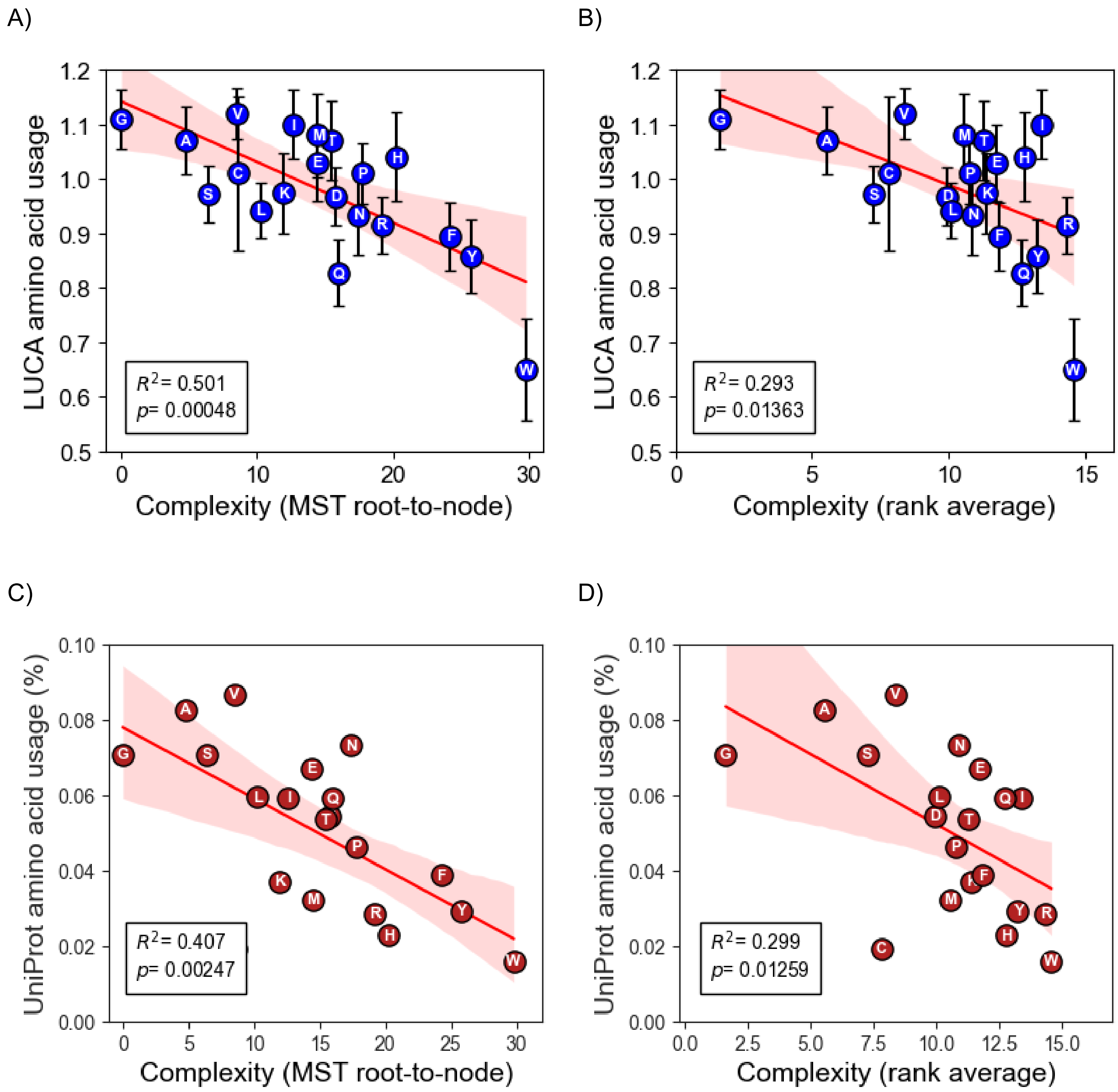

Figure S12. Correlation between molecular complexity, defined as root-to-node (A,C) and average rank (B,D) for LUCA amino acid usage [9] (A,B), and UniProt usage [11] (C,D). Error bars in (A,B) represent 95% confidence intervals derived from reported standard errors [9]. Red line shows linear regression fit, with shaded area indicating the 95% confidence interval of the fit.

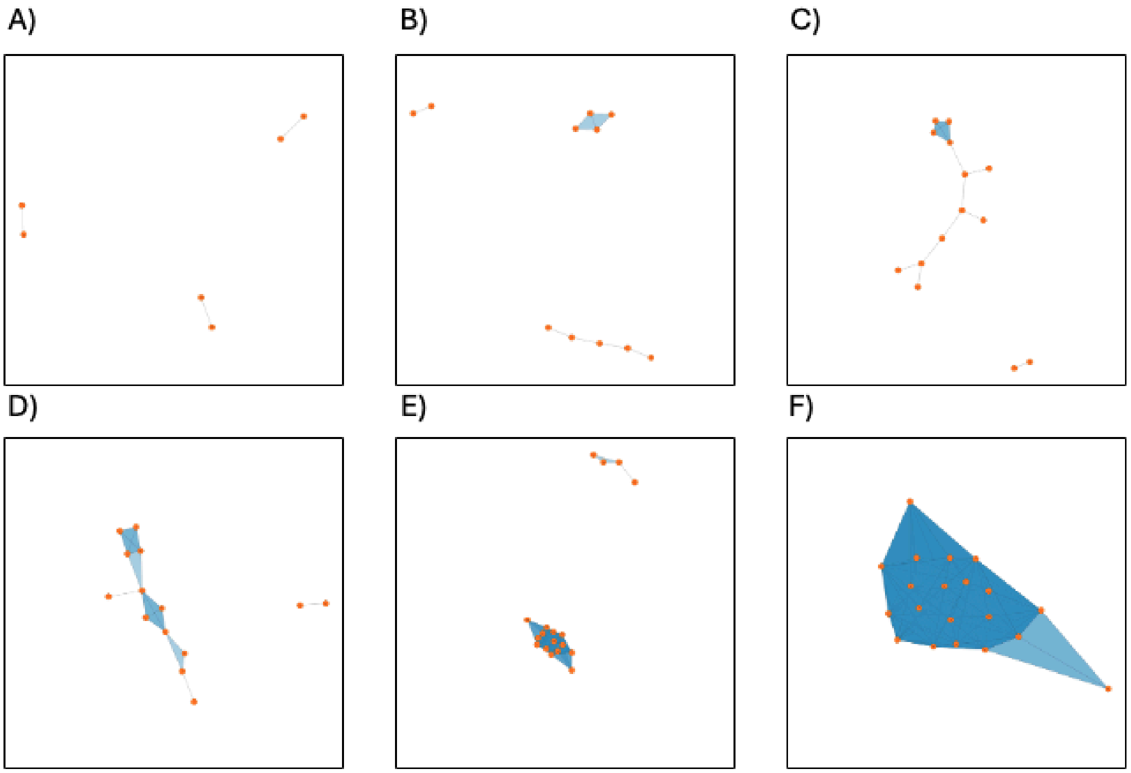

Figure S13. Plane-embedded filtered simplicial complex of amino acids at different filtration values: (A) Filtration = 0.2, (B) Filtration = 0.3, (C) Filtration = 0.35, (D) Filtration = 0.4, (E) Filtration = 0.6, (F) Filtration = 1, in units of one standard deviation. The filtration values represent increasing distance thresholds, progressively revealing the connectivity patterns between amino acids. At low filtration values, only a few local connections are present, while higher filtration values reveal more extensive connectivity, eventually forming a single connected component. This incremental analysis was used, in part, to identify the minimum threshold ($\epsilon = 1.0$) at which all nodes form a single connected component, ensuring the validity of the subsequent MST construction.

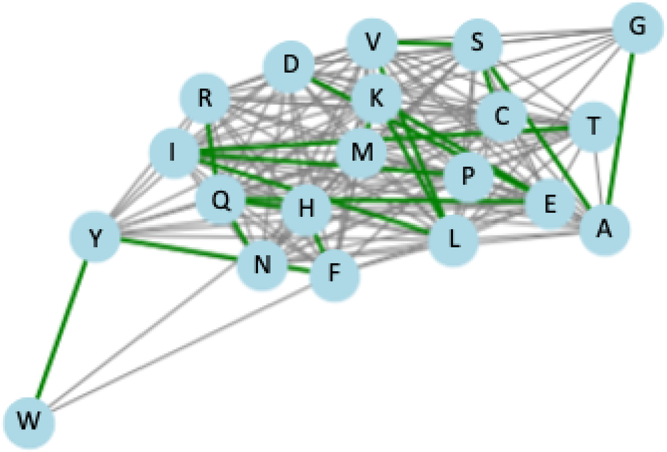

Figure S14. MST for amino acid nodes using connections derived from the filtered simplicial complex at the coarsest filtration value ($\epsilon = 1.0$). Green edges indicate the MST connections, corresponding to the edges shown in the main text figure (Fig. 4B). Gray edges represent additional connections present in the complete simplicial complex at the same filtration value.

Table S1. Summary of chemical graph-theory molecular complexity metrics included in this study, along with their sources, computational tools, and key features.

	Ref.	Source	Definition
Bertz/Hendrickson/Ihlenfeldt (BHI)	[12,13]	PubChem [14]	Integrates principles from chemical graph theory (molecular connectivity) and chemical information theory (heteroatom content).
Balaban index (BAL)	[15]	RDKit [16]	Quantifies molecular connectivity by considering both bond distances and branching patterns in a molecular graph.
Rucker's Walk Complexity (WCX)	[17]	Based on Molcomplex[1] [18]	Symmetry modified version of the Total Walk Count (TWC). Sums the number of all possible walks of varying lengths between atoms.
Proudfoot C_M index (PF)	[19]	Astrazeneca [20]	Determined by the number of unique paths and fraction of each type emanating from each atom.
Symmetry Modified Zagreb indices (SMM1, SMM2)	[21,22]	Mordred [23][2]	Symmetry modified version of the Zagreb indices (M1, M2). Based on the number of bonds emanating from each atom (SMM1) and each pair of consecutive atoms (SMM2).
Kappa shape indices (K1, K2, K3)	[24]	RDKit [16]	Compares a molecule to extreme shapes (fully linear or fully connected) with the same number of atoms.
Molecular index (MI)	[25]	AssemblyGo [25]	Quantifies the number of steps needed to assemble a molecule.

[1]Modified to exclude the ½ factor (see Note (23) in ref. [26]) and to account for symmetry [27].
[2]Modified to account for symmetry, as per ref. [22].

Table S2. Summary of chemical information-theory molecular complexity metrics included in this study, along with their sources, computational tools, and key features.

	Ref.	Source	Definition
Fraction of chiral carbons (FCC)	[28]	RDKit [16]	Measures the fraction of chiral centers in a molecule.
Fraction of sp3 hybridized carbons (Fsp3)	[29]	RDKit [16]	Measures the fraction of sp3 carbons in a molecule.
Normalized Spacial Score (nSPS)	[30]	Spacial-Score [30]	Calculated by summing atomic hybridization, stereoisomerism, ring participation, and squared neighbors for each heavy atom. Normalized by number of heavy atoms.
Bottcher Score per molar mass (NBS)	[31]	Values from [31]	Sums the information content of each atom's chemical environment, considering valence, bonds, stereochemistry, and chemical diversity, with corrections for molecular symmetry. Normalized per molar mass unit.
SMCM	[32]	Medchem[1]	Based on individual atoms electronegativity, bond types, SMARTS Patterns, ring size, fused rings, chiral centers, spiro carbon and bridged atoms.
Whitlock (WH)	[33]	Medchem[2]	Based on the number of rings, non-aromatic unsaturations and chiral centers.
Barone (BAR)	[34]	Medchem	Based on bond types, atomic diversity (in a limited sense) and ring size.
Minimal Graph Complexity (MGC)	[35]	Values from [35]	Translates the molecule into a sequence of symbols based on type of bonds and atoms. The sequence is compressed and converted into a numerical score.
Size/Complexity score (SCS)	[36]	Values from [37]	Assigns a score to each side chain based on the frequency of its component features relative to the set of 20 amino acids.
[1]Modified to count rings, fused rings, consecutive chiral carbons, and penalize pattern matches x2, as per ref. [32]<br>[2]Modified to count only non-aromatic rings, as per ref. [33]			

Table S3. Molecular complexity values for the 20 proteinogenic amino acids used in the analysis. Values are shown after all transformations, including log-scaling, normalization, and norm-based aggregation where applicable.

AA	BHI	BAL	logWCX	PF	NSMM	NK	MI	WH	BAR	SMCM	NBS	FCC	FSP3	nSPS	MGC	SCS
A	61.8	3.258	2.093	23.591	16.651	5.965	4	7	98	12.725	0.817	0.333	0.667	13.667	22.78	4.76
R	176	3.44	4.775	48.199	35.384	13.165	9	12	182	20.172	0.74	0.167	0.667	12.083	33.08	56.34
N	134	3.632	3.705	34.89	26.907	9.15	6	11	149	16.403	0.937	0.25	0.5	12.556	25.6	33.72
D	133	3.632	3.705	34.3	26.907	9.15	5	11	149	18.444	0.744	0.25	0.5	12.556	23.6	32.7
C	75.3	3.343	2.892	27.894	19.849	7.767	5	8	110	14.335	0.72	0.333	0.667	13.429	25.6	57.16
Q	146	3.491	4.068	40.104	29.732	10.429	6	11	158	18.403	0.888	0.2	0.6	12.5	27.9	37.48
E	145	3.491	4.068	39.515	29.732	10.429	5	11	158	20.444	0.714	0.2	0.6	12.5	25.9	36.48
G	42.9	2.847	1.944	17.761	12.207	5.861	3	5	66	8.725	0.519	0	0.5	7.4	17.6	1
H	151	2.499	4.607	46.359	38.184	8.61	9	9	224	14.974	1.017	0.167	0.333	12.818	36.3	58.7
I	103	3.716	3.794	39.353	27.586	9.438	6	9	148	21.725	0.774	0.333	0.833	16.778	29.3	16.04
L	101	3.502	3.705	38.455	26.907	9.937	6	7	128	18.725	0.692	0.167	0.833	13.778	28.6	16.04
K	106	3.215	4.03	43.159	28.32	11.557	6	8	137	20.731	0.711	0.167	0.833	13	26.2	30.14
M	97	3.266	3.664	36.416	25.495	10.819	7	8	128	13.71	0.713	0.2	0.8	13.111	28.9	64.68
F	153	2.585	4.978	50.978	41.11	9.851	9	7	239	22.727	0.617	0.111	0.222	12.417	36.6	44
P	103	2.35	3.36	36.186	28.04	6.039	6	11	152	18.582	0.905	0.2	0.8	28.25	24.78	31.8
S	72.6	3.343	2.892	27.894	19.849	7.201	4	8	110	14.772	0.829	0.333	0.667	13.429	25.6	17.86
T	93.3	3.638	3.173	33.591	24.459	8.074	5	10	142	19.772	1.009	0.5	0.75	17.375	28.78	21.62
W	245	2.323	6.634	66.885	59.464	10.591	11	8	341	31.352	0.883	0.091	0.182	12.867	44.6	73
Y	176	2.642	5.42	54.281	45.61	10.67	9	8	260	24.774	0.66	0.111	0.222	12.385	40.3	57
V	90.4	3.638	3.173	33.405	24.459	8.127	5	7	119	16.725	0.724	0.2	0.8	14	26.09	12.28

Table S4. Distance from each node (i.e. amino acid) to the root of the tree (i.e. less complex amino acid, glycine) for the combined sixteen metrics. Prebiotic amino acids are highlighted for comparison.

	Distance to root	Prebiotic consensus [7]
G	0	+
A	4.818	+
S	6.396	+
V	8.545	+
C	8.603	
L	10.262	+
K	11.937	
I	12.635	+
E	14.436	+
M	14.472	
T	15.499	+
D	15.811	+
Q	16.004	
N	17.414	
P	17.794	+
R	19.175	
H	20.244	
F	24.24	
Y	25.765	
W	29.79	

Table S5 Amino acids rank for each metric of molecular complexity.

<table>
<tr><th></th><th>1</th><th>2</th><th>3</th><th>4</th><th>5</th><th>6</th><th>7</th><th>8</th><th>9</th><th>10</th><th>11</th><th>12</th><th>13</th><th>14</th><th>15</th><th>16</th><th>17</th><th>18</th><th>19</th><th>20</th></tr>
<tr><td>BHI</td><td>G</td><td>A</td><td>S</td><td>C</td><td>V</td><td>T</td><td>M</td><td>L</td><td colspan="2">PI</td><td>K</td><td colspan="2">DN</td><td colspan="2">EQ</td><td>H</td><td>F</td><td colspan="2">YR</td><td>W</td></tr>
<tr><td>BAL</td><td>W</td><td>P</td><td>H</td><td>F</td><td>Y</td><td>G</td><td>K</td><td>A</td><td>M</td><td colspan="2">CS</td><td>R</td><td colspan="2">QE</td><td>L</td><td colspan="4">DNVT</td><td>I</td></tr>
<tr><td>logWCX</td><td>G</td><td>A</td><td colspan="2">SC</td><td colspan="2">TV</td><td>P</td><td>M</td><td colspan="3">DLN</td><td>I</td><td>K</td><td colspan="2">EQ</td><td>H</td><td>R</td><td>F</td><td>Y</td><td>W</td></tr>
<tr><td>PF</td><td>G</td><td>A</td><td colspan="2">SC</td><td colspan="2">VT</td><td>D</td><td>N</td><td colspan="2">PM</td><td>L</td><td colspan="2">IE</td><td>Q</td><td>K</td><td>H</td><td>R</td><td>F</td><td>Y</td><td>W</td></tr>
<tr><td>NSMM</td><td>G</td><td>A</td><td colspan="2">SC</td><td colspan="2">TV</td><td>M</td><td colspan="3">DLN</td><td>I</td><td>P</td><td>K</td><td colspan="2">EQ</td><td>R</td><td>H</td><td>F</td><td>Y</td><td>W</td></tr>
<tr><td>NK</td><td>G</td><td>A</td><td>P</td><td>S</td><td>C</td><td>T</td><td>V</td><td>H</td><td colspan="2">DN</td><td>I</td><td>F</td><td>L</td><td colspan="2">QE</td><td>W</td><td>Y</td><td>M</td><td>K</td><td>R</td></tr>
<tr><td>MI</td><td>G</td><td colspan="2">AS</td><td colspan="5">TEVCD</td><td colspan="6">LKPNQI</td><td>M</td><td colspan="4">HYFR</td><td>W</td></tr>
<tr><td>FCC</td><td>G</td><td>W</td><td colspan="2">YF</td><td colspan="4">RHLK</td><td colspan="5">VQEMP</td><td colspan="2">ND</td><td colspan="4">ACSI</td><td>T</td></tr>
<tr><td>FSP3</td><td>W</td><td colspan="2">YF</td><td>H</td><td colspan="3">NDG</td><td colspan="2">QE</td><td colspan="4">ARCS</td><td>T</td><td colspan="3">VMP</td><td colspan="3">KLI</td></tr>
<tr><td>nSPS</td><td>G</td><td>R</td><td colspan="6">YFQEND</td><td colspan="2">HW</td><td>K</td><td>M</td><td colspan="2">CS</td><td>A</td><td>L</td><td>V</td><td>I</td><td>T</td><td>P</td></tr>
<tr><td>NBS</td><td>G</td><td>F</td><td>Y</td><td>L</td><td colspan="3">KME</td><td>C</td><td>V</td><td>R</td><td>D</td><td>I</td><td>A</td><td>S</td><td>W</td><td>Q</td><td>P</td><td>N</td><td>T</td><td>H</td></tr>
<tr><td>SMCM</td><td>G</td><td>A</td><td>M</td><td>C</td><td>S</td><td>H</td><td>N</td><td>V</td><td colspan="2">QD</td><td>P</td><td>L</td><td>T</td><td>R</td><td>E</td><td>K</td><td>I</td><td>F</td><td>Y</td><td>W</td></tr>
<tr><td>WH</td><td>G</td><td colspan="4">AFLV</td><td colspan="6">YKMSWC</td><td colspan="2">HI</td><td>T</td><td colspan="5">QEDNP</td><td>R</td></tr>
<tr><td>BAR</td><td>G</td><td>A</td><td colspan="2">SC</td><td>V</td><td colspan="2">LM</td><td>K</td><td>T</td><td colspan="3">IND</td><td>P</td><td colspan="2">QE</td><td>R</td><td>H</td><td>F</td><td>Y</td><td>W</td></tr>
<tr><td>MGC</td><td>G</td><td>A</td><td>D</td><td>P</td><td colspan="3">SNC</td><td>E</td><td colspan="2">VK</td><td>Q</td><td>L</td><td colspan="2">TM</td><td>I</td><td>R</td><td>H</td><td>F</td><td>Y</td><td>W</td></tr>
<tr><td>SCS</td><td>G</td><td>A</td><td>V</td><td colspan="2">IL</td><td>S</td><td>T</td><td>K</td><td>P</td><td>D</td><td>N</td><td>E</td><td>Q</td><td>F</td><td>R</td><td colspan="2">YC</td><td>H</td><td>M</td><td>W</td></tr>
</table>

Table S6. Average rank and standard error (calculated as standard deviation divided by number of metrics). Prebiotic amino acids (from ref. [7]) are highlighted for comparison.

	Average rank	Standard error	Prebiotic consensus [7]
G	2.30	0.58	+
A	4.56	0.91	+
S	6.26	0.78	+
C	6.83	0.80	
V	7.48	0.77	+
T	9.78	0.99	+
D	9.87	0.68	+
P	9.98	1.04	+
L	10.00	0.77	+
N	10.44	0.72	
M	10.48	0.90	
K	11.83	0.80	
E	12.06	0.67	+
I	12.33	0.73	+
Q	12.87	0.60	
F	13.28	1.18	
H	13.39	1.01	
Y	14.96	1.17	
R	15.48	0.82	
W	15.81	1.33	

SI References

1. Kruskal JB. Multidimensional scaling by optimizing goodness of fit to a nonmetric hypothesis. Psychometrika. 1964;29: 1–27.
2. Young G, Householder AS. Discussion of a set of points in terms of their mutual distances. Psychometrika. 1938;3: 19–22.
3. Vietoris L. Über den höheren Zusammenhang kompakter Räume und eine Klasse von zusammenhangstreuen Abbildungen. Math Ann. 1927;97: 454–472.
4. Hausmann J-C. On the Vietoris-Rips complexes and a Cohomology Theory for metric spaces. In: Quinn F, editor. Prospects in Topology (AM-138). Princeton: Princeton University Press; 1996. pp. 175–188.
5. Prim RC. Shortest connection networks and some generalizations. Bell Syst Tech J. 1957;36: 1389–1401.
6. Trifonov EN. The triplet code from first principles. J Biomol Struct Dyn. 2004;22: 1–11.
7. Longo LM, Blaber M. Protein design at the interface of the pre-biotic and biotic worlds. Arch Biochem Biophys. 2012;526: 16–21.
8. Ali T, Borah C. Analysis of amino acids network based on mutation and base positions. Gene Rep. 2021;24: 101291.
9. Wehbi S, Wheeler A, Morel B, Manepalli N, Minh BQ, Lauretta DS, et al. Order of amino acid recruitment into the genetic code resolved by last universal common ancestor’s protein domains. Proc Natl Acad Sci U S A. 2024;121: e2410311121.
10. Trifonov EN. Consensus temporal order of amino acids and evolution of the triplet code. Gene. 2000;261: 139–151.
11. UniProt Consortium. UniProt: the Universal Protein Knowledgebase in 2025. Nucleic Acids Res. 2025;53: D609–D617.
12. Bertz SH. The first general index of molecular complexity. J Am Chem Soc. 1981;103: 3599–3601.
13. Hendrickson JB, Huang P, Toczko AG. Molecular complexity: a simplified formula adapted to individual atoms. J Chem Inf Comput Sci. 1987;27: 63–67.
14. Kim S, Chen J, Cheng T, Gindulyte A, He J, He S, et al. PubChem 2023 update. Nucleic Acids Res. 2023;51: D1373–D1380.
15. Balaban AT. Highly discriminating distance-based topological index. Chemical Physics Letters. 1982;89: 399–404.
16. Landrum G, Tosco P, Kelley B, Ric, sriniker, gedeck, et al. rdkit/rdkit: 2022_09_5 (Q3 2022) Release. Zenodo; 2023. doi:10.5281/zenodo.7671152
17. Ruecker G, Ruecker C. Counts of all walks as atomic and molecular descriptors. J Chem Inf Comput Sci. 1993;33: 683–695.
18. Wright BA, Okada T, Regni A, Luchini G, Sowndarya S S V, Chaisan N, et al. Molecular Complexity-Inspired Synthetic Strategies toward the Calyciphylline A-Type Alkaloids Himalensine A and Daphenylline. J Am Chem Soc. 2024;146: 33130–33148.
19. Proudfoot JR. A path based approach to assessing molecular complexity. Bioorganic & Medicinal Chemistry Letters. 2017;27: 2014–2017.
20. GitHub - AstraZeneca/molecular-complexity: Python implementation of the molecular complexity metric described by Proudfoot 2017 (http://dx.doi.org/10.1016/j.bmcl.2017.03.008). In: GitHub [Internet]. [cited 19 Jan 2025]. Available: https://github.com/AstraZeneca/molecular-complexity
21. Gutman I, Ruščić B, Trinajstić N, Wilcox CF Jr. Graph theory and molecular orbitals. XII. Acyclic

polyenes. J Chem Phys. 1975;62: 3399–3405.

22. Nikolic S, Tolic I, Trinajstic N, Baucic I. On the Zagreb indices as complexity indices. Croatica Chemica Acta. 2000;73: 909–921.
23. Moriwaki H, Tian Y-S, Kawashita N, Takagi T. Mordred: a molecular descriptor calculator. J Cheminform. 2018;10: 4.
24. Hall LH, Kier LB. The molecular connectivity chi indexes and kappa shape indexes in structure-property modeling. Reviews in Computational Chemistry. Hoboken, NJ, USA: John Wiley & Sons, Inc.; 2007. pp. 367–422.
25. Jirasek M, Sharma A, Bame JR, Mehr SHM, Bell N, Marshall SM, et al. Investigating and Quantifying Molecular Complexity Using Assembly Theory and Spectroscopy. ACS Central Science. 2024;10: 1054–1064.
26. Gutman I, Rücker C, Rücker G. On walks in molecular graphs. J Chem Inf Comput Sci. 2001;41: 739–745.
27. S. Nikolić, N. Trinajstić, I.M. Tolić, G. Rucker, C. Rucker. On molecular complexity indices. In: Bonchev D, Rouvray DH, editors. Complexity in Chemistry Introduction and Fundamentals. CRC Press; 2003.
28. Clemons PA, Bodycombe NE, Carrinski HA, Wilson JA, Shamji AF, Wagner BK, et al. Small molecules of different origins have distinct distributions of structural complexity that correlate with protein-binding profiles. Proc Natl Acad Sci U S A. 2010;107: 18787–18792.
29. Lovering F, Bikker J, Humblet C. Escape from Flatland: Increasing Saturation as an Approach to Improving Clinical Success. 2009;52: 6752–6756.
30. Krzyzanowski A, Pahl A, Grigalunas M, Waldmann H. Spacial Score─A Comprehensive Topological Indicator for Small-Molecule Complexity. J Med Chem. 2023;66: 12739–12750.
31. Böttcher T. An Additive Definition of Molecular Complexity. 2016;56: 462–470.
32. Allu TK, Oprea TI. Rapid evaluation of synthetic and molecular complexity for in silico chemistry. J Chem Inf Model. 2005;45: 1237–1243.
33. Whitlock HW. On the structure of total synthesis of complex natural products. J Org Chem. 1998;63: 7982–7989.
34. Barone R, Chanon M. A new and simple approach to chemical complexity. Application to the synthesis of natural products. J Chem Inf Comput Sci. 2001;41: 269–272.
35. Papentin F. On order and complexity. II. Application to chemical and biochemical structures. J Theor Biol. 1982;95: 225–245.
36. Dufton MJ. Genetic code synonym quotas and amino acid complexity: cutting the cost of proteins? J Theor Biol. 1997;187: 165–173.
37. Dufton MJ. Genetic code synonym quotas and amino acid complexity: cutting the cost of proteins? J Theor Biol. 1997;187: 165–173.
